# A PfRH5-Based Vaccine Is Efficacious against Heterologous Strain Blood-Stage *Plasmodium falciparum* Infection in *Aotus* Monkeys

**DOI:** 10.1016/j.chom.2014.11.017

**Published:** 2015-01-14

**Authors:** Alexander D. Douglas, G. Christian Baldeviano, Carmen M. Lucas, Luis A. Lugo-Roman, Cécile Crosnier, S. Josefin Bartholdson, Ababacar Diouf, Kazutoyo Miura, Lynn E. Lambert, Julio A. Ventocilla, Karina P. Leiva, Kathryn H. Milne, Joseph J. Illingworth, Alexandra J. Spencer, Kathryn A. Hjerrild, Daniel G.W. Alanine, Alison V. Turner, Jeromy T. Moorhead, Kimberly A. Edgel, Yimin Wu, Carole A. Long, Gavin J. Wright, Andrés G. Lescano, Simon J. Draper

**Affiliations:** 1 Jenner Institute, University of Oxford, Oxford OX3 7DQ, UK; 2 US Naval Medical Research Unit No. 6 (NAMRU-6), Lima, Peru; 3 Wellcome Trust Sanger Institute, Cambridge CB10 1HH, UK; 4 Laboratory of Malaria and Vector Research, NIAID/NIH, Rockville, MD 20852, USA; 5 Laboratory of Malaria Immunology and Vaccinology, NIAID/NIH, Rockville, MD 20852, USA

## Abstract

Antigenic diversity has posed a critical barrier to vaccine development against the pathogenic blood-stage infection of the human malaria parasite *Plasmodium falciparum*. To date, only strain-specific protection has been reported by trials of such vaccines in nonhuman primates. We recently showed that *P. falciparum* reticulocyte binding protein homolog 5 (PfRH5), a merozoite adhesin required for erythrocyte invasion, is highly susceptible to vaccine-inducible strain-transcending parasite-neutralizing antibody. In vivo efficacy of PfRH5-based vaccines has not previously been evaluated. Here, we demonstrate that PfRH5-based vaccines can protect *Aotus* monkeys against a virulent vaccine-heterologous *P. falciparum* challenge and show that such protection can be achieved by a human-compatible vaccine formulation. Protection was associated with anti-PfRH5 antibody concentration and in vitro parasite-neutralizing activity, supporting the use of this in vitro assay to predict the in vivo efficacy of future vaccine candidates. These data suggest that PfRH5-based vaccines have potential to achieve strain-transcending efficacy in humans.

## Highlights

• Vaccines based on the *P. falciparum* merozoite antigen PfRH5 were tested in *Aotus* monkeys
• PfRH5-based vaccines afforded protection against heterologous strains of *P. falciparum*
• Protection correlated with anti-PfRH5 IgG concentration and in vivo neutralization

## Graphical Abstract

**Figure:**
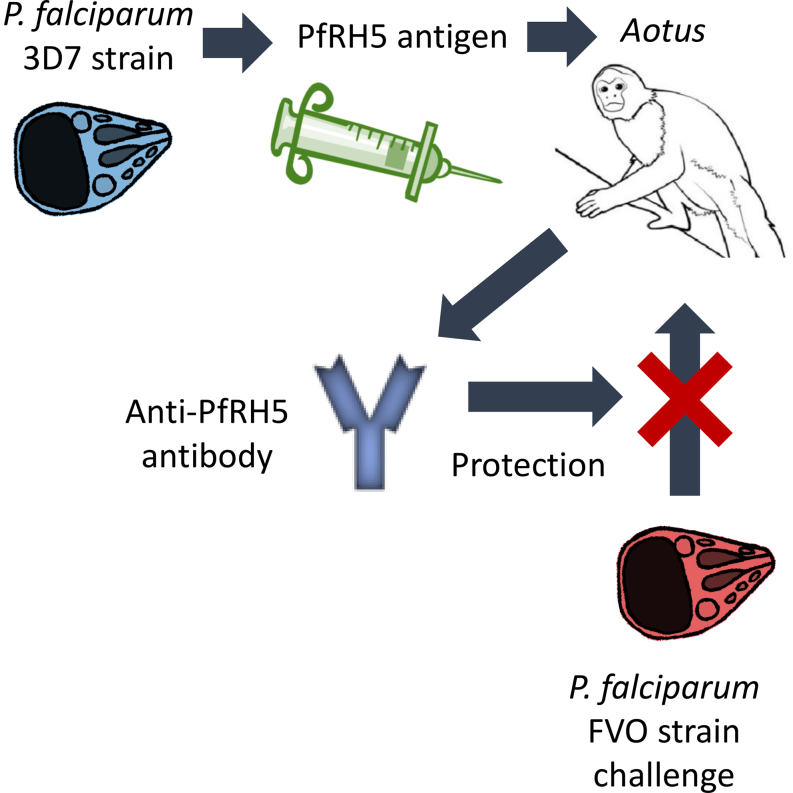

## Teaser

Antigenic diversity has hindered development of vaccines against the pathogenic blood-stage of *Plasmodium falciparum*. Douglas et al. demonstrate that human-compatible PfRH5-based vaccines can protect *Aotus* monkeys against vaccine-heterologous *P. falciparum* challenge. Protection correlated with anti-PfRH5 antibody concentration and parasite-neutralizing activity. PfRH5-based vaccines have potential to achieve strain-transcending efficacy in humans.

## Introduction

The development of a highly effective and deployable malaria vaccine remains an urgent priority for improving global public health. Despite recent strides in disease prevention and control, the *Plasmodium falciparum* human malaria parasite continues to exert a huge toll in terms of morbidity and mortality (Murray et al., 2012). The most advanced malaria subunit vaccine, a virus-like particle known as RTS,S, has shown only modest efficacy in young children in Phase III clinical trials (Agnandji et al., 2012), and thus new approaches are urgently needed (Moorthy et al., 2013).

RTS,S induces antibodies that reduce liver infection by the parasite (Foquet et al., 2014). An alternative and complementary strategy is to vaccinate against the subsequent blood-stage infection (which causes clinical disease and against which natural immunity is slowly acquired). Such a vaccine could prevent death and reduce incidence of disease, parasitemia, and onward transmission (Hill, 2011). However, despite 25 years of development, vaccine candidates targeting *P. falciparum*’s asexual blood stage have failed to overcome the challenge posed by the parasite’s antigenic diversity. Two of the most critical road blocks have included exceptionally high thresholds for protective levels of antibody against known target antigens, coupled with problematic levels of antigen polymorphism. To date, no vaccine candidate has overcome these hurdles to achieve in vivo protection in human clinical trials (Goodman and Draper, 2010; Thera et al., 2011). In previous nonhuman primate (NHP) studies (which provide the only opportunity to study the effect of vaccines against an uninterrupted *P. falciparum* blood-stage infection), blood-stage vaccine candidates have proven protective only against vaccine-homologous parasite lines, and only when administered with non-human-compatible adjuvants (Dutta et al., 2009; Lyon et al., 2008).

*P. falciparum* reticulocyte-binding protein homolog 5 (PfRH5) is a recently identified merozoite protein, secreted from the apical organelles of the parasite during the red blood cell (RBC) invasion process (Baum et al., 2009). In vitro data have identified PfRH5 as the highest priority target in the blood-stage malaria vaccine field for over a decade (Douglas et al., 2011). Antibodies induced by PfRH5 vaccination of mice and rabbits overcome the two major difficulties outlined above: (i) antibodies can block erythrocyte invasion to high efficiency (with lower EC_50_ in terms of μg/ml antigen-specific antibody than against all other known antigens) (Douglas et al., 2014; Miura et al., 2009; Williams et al., 2012) and (ii) most importantly, these antibodies cross-inhibit all *P. falciparum* lines and field isolates tested to date (Bustamante et al., 2013; Douglas et al., 2011; Reddy et al., 2014; Williams et al., 2012).

The PfRH5 protein is now known to mediate a critical nonredundant interaction with the human RBC surface protein basigin during invasion (Crosnier et al., 2011). The *PfRH5* gene is also refractory to genetic deletion (Baum et al., 2009; Hayton et al., 2008), unlike many other blood-stage antigens, confirming the essential nature of its function. In the context of natural infection, PfRH5 does not appear to be a dominant target of naturally acquired immune responses in endemic populations (Douglas et al., 2011; Tran et al., 2014; Villasis et al., 2012), but when detected, such antibody responses correlate with protective clinical outcome (Tran et al., 2014), and affinity-purified anti-PfRH5 human antibodies can neutralize parasites in vitro (Patel et al., 2013; Tran et al., 2014). The high degree of PfRH5 sequence conservation is thus associated with low-level natural immune pressure, but also functional constraints linked to basigin binding. Importantly, it has been shown that minimal amino acid substitutions in PfRH5 account for loss of basigin binding and/or host RBC tropism (linked to binding basigin orthologs from other species), suggesting the antigen may not easily escape vaccine-induced immune pressure (Hayton et al., 2008, 2013; Wanaguru et al., 2013). However, to date, no study has assessed the protective efficacy of PfRH5-based vaccines in vivo, and it remains unclear whether the encouraging observations made in vitro using an assay of parasite neutralization will translate into biologically relevant antiparasitic activity. This question is of particular importance, given the current lack of a clear correlate of vaccine efficacy against blood-stage infection in humans (Duncan et al., 2012) and the need to design improved strain-transcending malaria vaccines that can be progressed to clinical development.

In this study, we quantitatively assessed the immunogenicity of PfRH5-based vaccines delivered to *Aotus* monkeys by three different immunization regimens, including protein-in-adjuvant formulations (de Cassan et al., 2011) and an adenovirus/poxvirus vectored platform previously optimized for Phase I/IIa clinical development (Draper et al., 2008; Sheehy et al., 2012). We also evaluated the protective efficacy of these vaccines against a stringent vaccine-heterologous *P. falciparum* challenge (Stowers and Miller, 2001). This study enabled us to monitor the ability of PfRH5-based vaccines to both control and clear a virulent blood-stage infection. We report that significant protection against challenge with heterologous-strain blood-stage *P. falciparum* can be achieved in vivo by these vaccines, including when using the human-compatible viral vectored delivery platform. This protection was associated with anti-PfRH5 antibody concentration and parasite-neutralizing activity, supporting the use of this assay to predict the in vivo efficacy of future vaccine candidates. These results suggest that PfRH5-based vaccines have the potential to achieve strain-transcending efficacy in humans.

## Results

### Evaluation of PfRH5 Vaccine Efficacy in *Aotus* Monkeys

**Figure 1:**
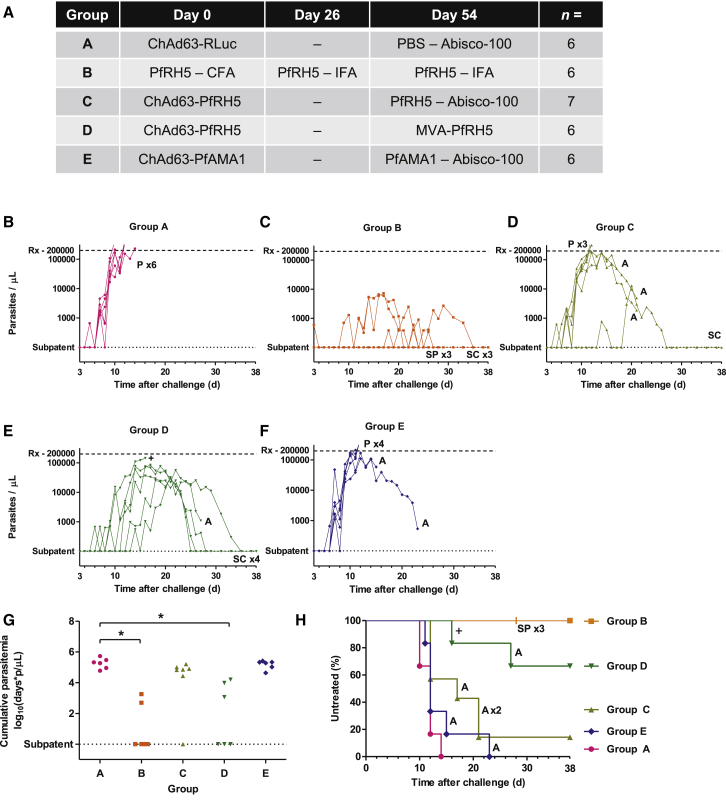
PfRH5 Vaccines Protect against *P. falciparum* Challenge (A) Vaccination regimes. Immunizations were performed by the intramuscular route on days 0 and 54, with the exception of those containing Freund’s adjuvant, which were given subcutaneously on days 0, 26, and 54. Doses used were 5 × 10^9^ infectious units (ifu) for ChAd63 vaccines, 2 × 10^8^ plaque-forming units (pfu) for MVA, 50 μg for protein vaccines, 250 μl for Freund’s adjuvant, and 48 μg for Abisco-100. (B–F) Time course of parasitemia for individual animals in Group A (B), Group B (C), Group C (D), Group D (E), and Group E (F). Upper horizontal dashed line indicates the 200,000 p/μl threshold for initiation of antimalarial treatment (Rx) because of hyperparasitemia; lower horizontal dotted line indicates absence of thin-film detectable parasites. P indicates treatment due to hyperparasitemia; A indicates treatment due to anemia; SPx3′ at day 28 in Group B indicates cessation of follow-up of three animals that had been microscopically subpatent since day 4; SC indicates self-cure in animals that had experienced sustained parasitemia; + indicates a single animal found dead on day 16; occasional unexpected deaths have previously been recorded among *Aotus* both before and during *P. falciparum* challenge (Darko et al., 2005; Hisaeda et al., 2002; Singh et al., 2006). (G) Cumulative parasitemia up to day 10, the first day on which an animal was treated. ^∗^p < 0.01 versus Group A by Mann-Whitney test performed with Bonferroni correction for multiple comparison (this was the prespecified primary analysis for Groups C–E; secondary analysis for Group B). (H) Kaplan-Meier plot of percentage untreated survival by group. Symbols are as in (B)–(F). Comparing time to treatment in each group to Group A was done by Mann-Whitney test with Bonferroni correction for multiple comparison, p = 0.02 for each of Groups B and D. See also Figures S1 and S5 and Table S1.

31 *Aotus nancymaae* monkeys were randomized to groups that received protein-in-adjuvant and/or viral vectored vaccination regimes targeting either *P. falciparum* RH5 or apical membrane antigen 1 (PfAMA1), a well-studied comparator antigen that elicits strain-specific antibodies (Dutta et al., 2009; Remarque et al., 2008; Thera et al., 2011) (Figure 1A). The PfRH5 protein immunogen was pure (Figure S1A) and shown to be correctly folded by demonstration of binding to its receptor, basigin (Crosnier et al., 2011) (Figure S1B). Group A received sham vaccines, chimpanzee adenovirus serotype 63 (ChAd63) expressing *Renilla* luciferase (RLuc) prime, PBS with Abisco-100 adjuvant boost; Group B received PfRH5 protein with complete or incomplete Freund’s adjuvant (CFA, IFA); Group C received ChAd63 expressing PfRH5 prime, PfRH5 protein with Abisco-100 boost; Group D received ChAd63-PfRH5 prime, modified vaccinia virus Ankara (MVA) expressing PfRH5 boost; and Group E received ChAd63-PfAMA1 prime, PfAMA1 protein with Abisco-100 boost. The ChAd63-MVA vaccine delivery platform used here has now been progressed to human clinical testing for a wide variety of difficult disease targets, including malaria, HIV-1, and hepatitis C virus (de Cassan and Draper, 2013; Draper and Heeney, 2010), while the use of mixed-modality adenoviral priming-protein-boost regimens has shown promise in small animals as well as initial clinical studies (de Cassan et al., 2011; Draper et al., 2010) (Hodgson et al., 2014). In the case of this study, the PfRH5 vaccines encoded the 3D7 allele of the antigen, while for PfAMA1 the ChAd63 vector expressed two alleles of the antigen (3D7 and FVO), and FVO allele PfAMA1 protein was used for the boost. The Group A sham-vaccinated animals served as protocol-specified infectivity controls in order to confirm consistent infection by the FVO parasite inoculum and its appropriate adaptation to growth in *Aotus*.

To evaluate the protective efficacy of the vaccines, animals were challenged 15 days after the final vaccination by intravenous administration of 10^4^ PfRH5-vaccine-heterologous FVO strain *P. falciparum* infected red blood cells (iRBC) taken from a donor monkey. The parasitemia (Figures 1B–1F) and hematocrit (Hct) (Figures S1C–S1G) in the challenged animals were monitored over time. Challenge infection with this parasite line has proven highly virulent in *Aotus nancymaae* over the course of numerous studies, requiring treatment in all control animals administered complete Freund’s adjuvant without a blood-stage vaccine antigen (n = 55, Table S1 and Supplemental Information). In contrast, none of the animals immunized here with PfRH5 protein in Freund’s adjuvant (Group B) required treatment. Efficacy in this group was significant, both comparing treatment status versus adjuvant-matched historical controls (the protocol-specified primary analysis for this group; Kendall’s τ_B_ = 0.703, p < 0.001 versus historical controls) and comparing log_10_ cumulative parasitemia (LCP) up to the first day on which an animal was treated (day 10) versus Group A in the current study (p = 0.002 by Mann-Whitney test, Figure 1G). No parasites were seen by thin-film microscopy at any point in two of the animals, with only a single parasite seen on one occasion in a third animal; the remaining three animals self-cured after periods of microscopically patent parasitemia at levels <10,000 parasites/μl (p/μl) (Figure 1C). To our knowledge, such robust protection has not been observed even after vaccine-homologous challenge of *Aotus* immunized with *P. falciparum* AMA1, merozoite surface protein 1 (PfMSP1), PfMSP3, or the erythrocyte binding antigen 175 kDa (PfEBA175) formulated with Freund’s adjuvant (Hisaeda et al., 2002; Jones et al., 2001; Stowers et al., 2001, 2002). Here, the PfRH5 antigen of the challenge strain, FVO, differed at four amino acid loci from the 3D7 clone upon which the vaccine was based (Hayton et al., 2008). No greater level of divergence from the 3D7 antigen was identified among 227 field parasite strains recently sequenced (Manske et al., 2012; Williams et al., 2012).

Significant vaccine efficacy was also observed in the animals immunized with human-compatible ChAd63-MVA PfRH5 vaccines (Group D), as judged using the prespecified primary endpoint for Groups C–E of LCP up to day 10 as compared to Group A (Figure 1G; p = 0.007 by Mann-Whitney test with Bonferroni correction for multiple comparison). Similar to Freund’s adjuvant, there are abundant pre-existing data across multiple species to demonstrate that there is no nonspecific effect of the viral vector vaccines used in Group D upon the rate of *Plasmodium* blood-stage infection, including for *P. falciparum* in malaria-challenged humans as compared to unvaccinated infectivity controls (Ewer et al., 2013; Sheehy et al., 2012), as well as in three rodent malaria species: *P. yoelii*, *P. berghei*, or *P. chabaudi* (Biswas et al., 2012; Goodman et al., 2013). There was also no nonspecific effect of vaccination with COPAK (a poxvirus similar to the MVA) upon the course of *P. knowlesi* parasitemia in rhesus macaques (Weiss et al., 2007). However, as is typical in this model, a number of animals that developed but controlled relatively high-level parasitemia in Groups C and D subsequently required treatment due to anemia (Figures 1H and S1C–S1G), the severity and timing of which correlated with the level and timing of peak parasitemia (Figures S2H–S2J). Most importantly, unlike previous trials of a human-compatible blood-stage vaccine delivery platform in this model, self-cure of infection without severe anemia was observed in 4/6 animals in the ChAd63-MVA PfRH5 group and 1/7 in the ChAd63-Protein PfRH5 group (Figures 1D, 1E, and 1H). The four self-curing animals in the ChAd63-MVA PfRH5 group experienced median peak parasite density of 48,000 p/μl (median peak percentage parasitemia 0.9%) and were afebrile and clinically well throughout.

### Immunologic Correlates of Protection

The rationale for the development of these PfRH5 vaccines was the observation that PfRH5-specific antibodies could achieve high levels of parasite-neutralizing in vitro growth inhibitory activity (GIA) (Bustamante et al., 2013; Douglas et al., 2011; Williams et al., 2012). We therefore hypothesized prior to the trial that protection achieved by PfRH5 vaccines would be associated with anti-PfRH5 antibody concentrations and in vitro GIA, but not necessarily with cellular responses against PfRH5. To test this hypothesis, we initially assessed humoral and cellular immune responses by ELISA, interferon-γ (IFN-γ) ELISpot, and intracellular cytokine staining (ICS) assays.

**Figure 2:**
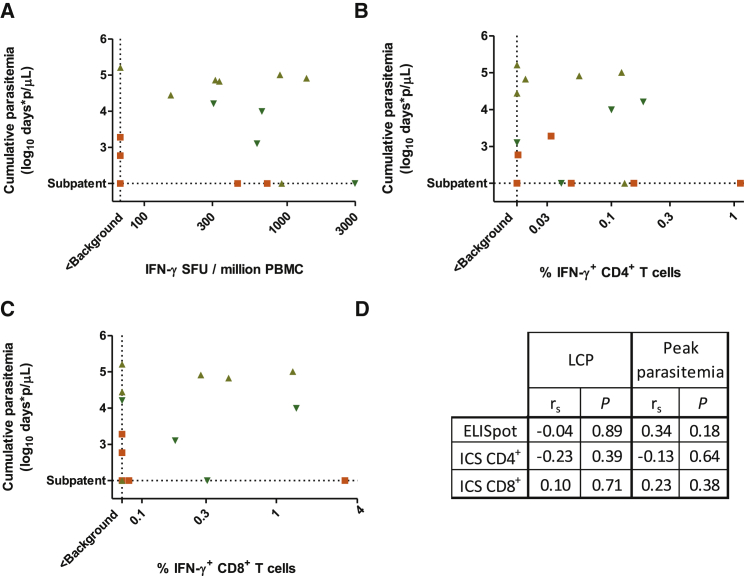
Lack of Relationship between ELISpot and ICS Responses and Challenge Outcome (A–C) Relationships between challenge outcome (LCP) and PfRH5-specific ELISpot responses (A), IFN-γ^+^ CD4^+^ T cell responses (B), and IFN-γ^+^ CD8^+^ T cell responses (C) in PfRH5-vaccinated animals (n = 17 for ELISpot, n = 16 for ICS; Groups B, C, D, excluding the two animals for which ELISpot results were not obtained and the three animals for which ICS results were not obtained). All panels plot responses after subtraction of responses in negative control unstimulated wells. Dashed line marked “< Background” indicates responses of less than the mean plus three standard deviations (SD) of the apparent response in Group A animals. (D) Lack of statistically significant Spearman’s rank correlation between ELISpot and ICS-measured immunological parameters and outcome parameters (LCP or peak parasitemia; no Bonferroni correction was made). Measurements that did not exceed background (defined as above) were treated as tied observations. See also Figure S2.

Antigen-specific T cell responses to a subunit vaccine have rarely been measured in *Aotus*, but we were able to detect PfRH5 and PfAMA1-specific IFN-γ-producing PBMCs by ELISpot (Figures S2A–S2C) and resolve these into CD4^+^ and CD8^+^ T cells by ICS (Figures S2D–S2G). There was no correlation between ELISpot or ICS responses and LCP or peak parasitemia (Figures 2A–2D).

**Figure 3:**
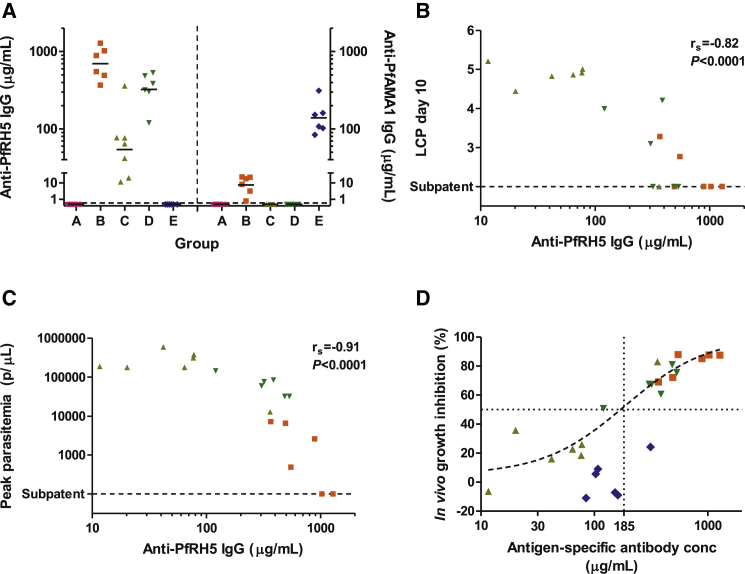
Protection Is Associated with Prechallenge Anti-PfRH5 Antibody Concentration (A) Plasma were tested by ELISA on the day of challenge (DoC, day 69) for anti-PfRH5 (3D7) allele (left y axis) or anti-PfAMA1 (FVO) allele (right y axis) total IgG responses. Individual responses and geometric mean are shown for each group. Arbitrary ELISA units were converted to μg/ml concentrations following definition of a conversion factor by CFCA (Figure S3). (B–D) Relationships between DoC plasma antibody concentration and challenge outcome. For the 19 PfRH5-vaccinated animals, Spearman’s rank correlation coefficient (r_s_) and p value are shown for the relationship of anti-PfRH5 total IgG concentration on DoC with the primary endpoint (LCP) (B) and peak parasitemia (C). (D) The relationship between IVGI and antigen-specific antibody concentration (anti-PfRH5 in the 19 PfRH5 vaccinated animals, and anti-PfAMA1 for the 6 PfAMA1-vaccinated animals). A nonlinear regression curve was fitted to the points from the PfRH5-vaccinated animals and used to estimate IVGI EC_50_, as reported in the text. There were insufficient data for curve fitting to the PfAMA1-vaccinated animals. See also Figures S3 and S4.

The vaccines also induced substantial PfRH5-specific antibody responses, as measured by ELISA with conversion into absolute antigen-specific antibody concentrations achieved via surface plasmon resonance (SPR) calibration-free concentration analysis (CFCA) (Williams et al., 2012) (Figures 3A, S3, and S4A–S4F). Geometric mean day-of-challenge (DoC) PfRH5-specific antibody concentrations achieved were 700 μg/ml in Group B (PfRH5 protein in Freund’s adjuvant), 54 μg/ml in Group C (ChAd63-Protein PfRH5), and 320 μg/ml in Group D (ChAd63-MVA PfRH5); the corresponding geometric mean anti-PfAMA1 (FVO allele)-specific antibody concentration in Group E was 140 μg/ml. There was a strong and statistically significant correlation between anti-PfRH5 antibody concentration and challenge outcome (Figures 3B–3D). These correlations were maintained when the analysis was conducted among animals *within* Groups B and D (Figures S4G and S4H). Given that these animals serve as matched controls for each other within a group, such a correlation would be unlikely if protection was attributable to a nonspecific effect of the adjuvant or the viral vaccine vector.

In vivo growth inhibition was also calculated (IVGI; the percentage reduction in the parasite multiplication rate (PMR) in each animal relative to the mean in the control group, as has been previously described; Mahdi Abdel Hamid et al., 2011). Using nonlinear least-squares regression, the concentration of anti-PfRH5 immunoglobulin G (IgG) required to achieve 50% IVGI (IVGI EC_50_) was estimated at 185 μg/ml (95% CI 100–330 μg/ml, using data from Groups B–D). The relationship between PfAMA1-specific antibody concentration and IVGI was suggestive of a higher IVGI EC_50_ for anti-PfAMA1 IgG than for anti-PfRH5 IgG (Figure 3D). These in vivo growth inhibition data are thus in line with previous in vitro GIA data using purified antigen-specific anti-PfRH5 and anti-PfAMA1 IgG from rabbits and humans (Miura et al., 2009; Tran et al., 2014), suggesting that parasites are quantitatively more susceptible to anti-PfRH5 than to anti-PfAMA1 antibodies.

**Figure 4:**
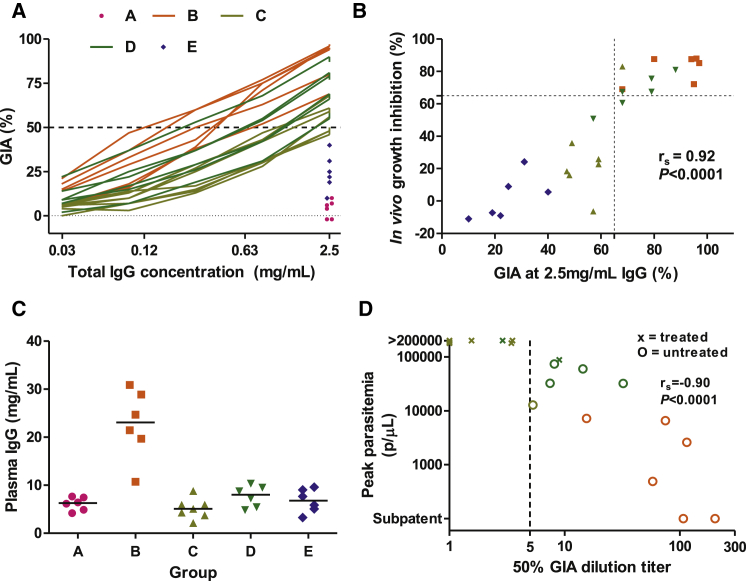
Attainment of 50% GIA at a Subphysiological IgG Concentration Predicts Protection (A) The in vitro GIA of purified IgG (DoC time point) was assessed against FVO strain parasites for all animals at a fixed concentration of 2.5 mg/ml. Percent GIA is reported following a single cycle of parasite growth. For animals in Groups B, C, and D, the assays were repeated with a dilution series of the purified IgG. The results for each individual animal are shown. (B) Prespecified primary analysis for an immunological correlate of protection: relationship between percent GIA using 2.5 mg/ml purified IgG in the assay and the percent IVGI modeled from the parasitemia data. Across all animals (n = 31), Spearman’s r_s_ = 0.86, p < 0.0001; among noncontrol vaccinated animals only (Groups B–E, n = 25, as shown), r_s_ = 0.92, p < 0.0001. The dashed lines identify animals in the top right quadrant that did not require treatment following challenge. GIA was predictive of outcome independent of group allocation (p = 0.004 by likelihood-ratio test comparing a bivariate model relating IVGI with group versus a multivariate model relating IVGI with group plus GIA at 2.5 mg/ml). (C) The plasma concentration of IgG in mg/ml was assayed at the DoC time point. Individual results and group medians are reported. (D) GIA_50_ titer was calculated for Groups B, C, and D (by dividing the plasma total IgG concentration by the total IgG GIA EC_50_) and is plotted against peak parasitemia. Three animals in Group C, which all required treatment, did not achieve 50% GIA at 2.5 mg/ml and were assigned tied GIA_50_ titers of 1. Spearman’s rank correlation coefficient is shown. See also Figure S5.

We subsequently assessed the functional ability of the vaccine-induced antibody responses to neutralize parasites in the GIA assay. The ability of a range of concentrations of protein G-purified total IgG to neutralize FVO (challenge-strain) parasites was assessed (Figure 4A). There was a strong and statistically significant relationship between GIA at 2.5 mg/ml total IgG and IVGI, the protocol-specified primary analysis for an immunological correlate of protection (Figure 4B). Immunological correlates of vaccine-induced protection against other pathogens are commonly the attainment of a particular level of in vitro activity at a certain serum dilution titer (Plotkin, 2010). Here, we observed that the total plasma IgG concentration of animals immunized with different regimes differed substantially (Figure 4C). We therefore calculated a GIA_50_ titer, defined as the dilution factor from the plasma IgG concentration to the IgG concentration achieving 50% GIA. GIA_50_ titer was closely associated with challenge outcome (Figure 4D), with attainment of a GIA_50_ titer exceeding five appearing predictive of untreated survival.

### Impact of Infection on Vaccine-Induced Responses

**Figure 5:**
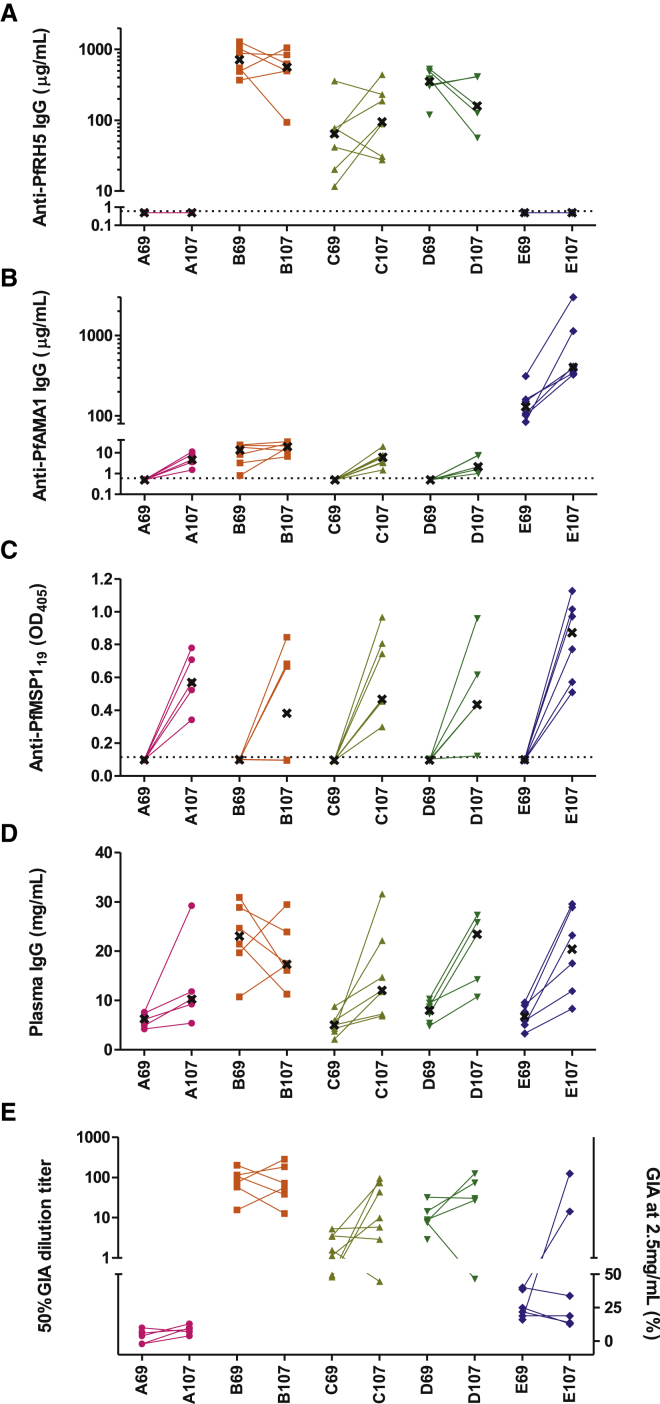
Post-Challenge Measures of Immunogenicity (A–E) Plasma antigen-specific total IgG responses and GIA were also assessed in the post-challenge period. Individual responses are shown and linked for each animal in each group at the DoC time point (day 69) and at the end of the challenge monitoring period (day 107, 38 days post-challenge). Median responses are indicated, where possible, by a black cross. Anti-PfRH5 (A), anti-PfAMA1 (B), and anti-PfMSP1_19_ (C) ELISA data are shown. (D) Plasma IgG concentration in mg/ml. (E) GIA was assessed as in Figure 4, initially testing all samples at 2.5 mg/ml purified IgG. If >50% GIA was observed, samples were diluted in the assay, and the GIA_50_ titer was calculated. For each animal at each time point, GIA_50_ titer is shown on the top left y axis (where possible to calculate); otherwise, the percent GIA at 2.5 mg/ml is indicated on the lower right y axis. See also Figure S5.

We also monitored immune responses in the period following the challenge infection. Consistent with the poor immunogenicity of PfRH5 in the context of natural infection (Douglas et al., 2011; Tran et al., 2014; Villasis et al., 2012), the effect of parasite exposure on PfRH5-specific antibody levels was variable (Figure 5A) when assessing the day of challenge (day 69) versus the end of the challenge follow-up period (day 107). In contrast, median anti-PfAMA1 IgG levels increased across all the groups, but most notably in Group E (Figure 5B). Nearly every animal experiencing a patent infection seroconverted to the 19 kDa C terminus of PfMSP1 (PfMSP1_19_; Figure 5C), consistent with the abundant and immunodominant nature of this merozoite surface antigen, supported by similar data from controlled human malaria infection (CHMI) studies in malaria-naive adult volunteers (Elias et al., 2014). Changes in GIA measured at a constant total IgG concentration of 2.5 mg/ml in the same period were small, with no significant change in PfRH5-vaccinated, PfAMA1-vaccinated, or sham-vaccinated animals (median changes of −7%, −3%, and 6%; p = 0.21, 0.81, and 0.14, respectively, by Wilcoxon signed-rank test). Nonetheless, GIA_50_ titers increased substantially in many cases, due to increases in total plasma IgG concentration after parasite exposure (Figures 5D and 5E). Follow-up of animals to day 230 (161 days after challenge) demonstrated that, in several animals, anti-PfRH5 antibody concentrations were maintained at levels in excess of the IVGI EC_50_ for a number of months after challenge (Figure S5A).

## Discussion

Overall, these results demonstrate that PfRH5-based vaccines have the potential to overcome the shortcomings of previous blood-stage vaccines against *P. falciparum* (Wright and Rayner, 2014). The attainment of cross-strain protection in vivo by an antigen selected on the basis of its ability to induce cross-strain GIA in vitro strongly hints that the relationship between GIA and protection is causal, encouraging the continued use of the assay for candidate vaccine selection. Our findings are also consistent with previous data linking protection with the attainment of 60% GIA against the challenge strain at 2.5 mg/ml total IgG, suggesting that the quantitative relationship between GIA and protection may be roughly similar for different antigens (Singh et al., 2006). Importantly, the efficacy observed here would also have been readily detectable in a Phase IIa CHMI clinical trial (Figures S5B and S5C). These data thus support the assertion that an efficacious blood-stage vaccine candidate should be able to demonstrate in vivo biological effects in CHMI trials prior to field trials (Sheehy et al., 2013).

Protection in this study appeared to be mediated by preformed anti-PfRH5 antibody present at the time of infection, as distinct from a recall response against PfRH5 after challenge. Antigen-specific antibody concentrations exceeding 100 μg/ml have been attained in humans by other malaria vaccines including RTS,S and those targeting PfAMA1 (Kester et al., 2009; Spring et al., 2009), but long-term maintenance of such high-level responses may be challenging, particularly if *P. falciparum* infection does not appreciably boost vaccine-induced anti-PfRH5 responses. We have recently demonstrated that antibodies of other specificities can act synergistically with anti-PfRH5 antibodies in GIA assays, thus supporting an ongoing strategy to achieve protection with substantially lower and more easily maintained antibody concentrations (Williams et al., 2012).

Like the functionally critical surface proteins of other challenging vaccine targets, such as HIV-1 and influenza virus, the immunodominant antigens of *Plasmodium* spp. are highly variable (Riley and Stewart, 2013). PfRH5 is also functionally critical, but analogous to the pre-erythrocytic malaria antigen circumsporozoite protein (the basis of the RTS,S vaccine), the response to it in the context of infection is unlikely to be of sufficient magnitude to be a substantial contributor to natural immunity (Douglas et al., 2011; Murungi et al., 2013; Tran et al., 2014). Although this immune evasion strategy is clearly successful in permitting *P. falciparum* to establish repeated and chronic infections, it has left a conserved whole-protein target that appears more susceptible to subunit vaccination than the conserved epitopes presented by HIV-1 and influenza.

The clinical implications of these data for PfRH5-based vaccines remain unknown for now and will require clinical trials to assess immunogenicity and efficacy in humans (Figure S5D). However, given the attainment of an unprecedented level of protection in a stringent model, this study has clearly demonstrated that the problem of interstrain blood-stage antigen variation is tractable. It is worth noting that the FVO strain of *P. falciparum* has been selected for rapid growth and virulence in *Aotus* monkeys; as a result, it is considerably more virulent in these animals (essentially universally fatal in untreated animals) than is the case for *P. falciparum* in humans (in which the probability of severe disease is thought not to exceed 15% per episode even in malaria-naive infants) (Collins et al., 1994; Gupta et al., 1999). Moreover, having defined two significant correlates of protection, it will now be possible to rationally improve PfRH5-based immunogens and their delivery in order to maximize functional antiparasitic immunity. The optimization of PfRH5 vaccine formulations to achieve and maintain the highest possible levels of antibody will also be a major focus of human clinical trials that should commence within the coming year, supported by a greater weight of preclinical evidence than any previous blood-stage vaccine candidate. In summary, this study provides the initial proof of concept in NHPs that the development of a strain-transcending blood-stage vaccine against *P. falciparum* is possible and provides important insight into a correlate of protection against the human malaria parasite.

## Experimental Procedures

Full experimental methods are provided in detail in the Supplemental Information.

### Vaccines

All PfRH5 vaccines were based upon the *P. falciparum* 3D7 clone sequence. The production of recombinant MVA expressing full-length PfRH5 has previously been described (Douglas et al., 2011). For the current study, a ChAd63 vector expressing the same PfRH5 transgene was produced using previously described methods (Goodman et al., 2010). The production of the ChAd63 and protein PfAMA1 (FVO allele) vaccines have been reported elsewhere (Biswas et al., 2011; Kennedy et al., 2002). The PfRH5 3D7 protein was expressed and tested for basigin binding essentially as described (Crosnier et al., 2011).

### Animals, Immunizations, Challenge, and Sample Collection

Adult female owl monkeys (*Aotus nancymaae*) were housed at the US Naval Medical Research Unit No. 6 (NAMRU-6). Randomization to groups was stratified by pretrial weight. All immunizations were administered under ketamine anesthesia and performed by the intramuscular route (into the caudal quadriceps), with the exception of those containing Freund’s adjuvant, which were given subcutaneously (into the interscapular area). The study protocol was approved by NAMRU-6’s Institutional Animal Care and Use Committee (protocol number NAMRU-6 11-12), the Department of the Navy Bureau of Medicine and Surgery (NRD-748), the University of Oxford Animal Care and Ethical Review Committee, and the Institut Nacional de Recursos Naturales (INRENA) at the Peruvian Ministry of Agriculture.

15 days after the final vaccination, animals were challenged intravenously with 10^4^ FVO-strain *P. falciparum* iRBC taken from a donor monkey, as previously described (Stowers et al., 2001). From day 72, daily thin-film parasitemia quantification and alternate-day Hct measurements were conducted. Animals were treated (i) when parasite density reached ≥200,000/μl, (ii) when Hct fell to ≤25%, (iii) upon reaching challenge day 28 (C+28) if no parasites had been seen in the preceding week, or (iv) upon reaching C+38 (study day 107). Blood samples for immunological assays were collected from all animals’ saphenous veins under ketamine anesthesia. EDTA-anticoagulated blood was prepared using standard methods to obtain plasma and PBMC.

### Cellular Immune Assays

Ex vivo IFN-γ ELISpot and ICS were performed essentially as previously described (Draper et al., 2010). Assays used frozen PBMC and pools of PfRH5 and PfAMA1 peptides.

### ELISA, CFCA, and GIA

PfAMA1 ELISAs used the same recombinant PfAMA1 FVO protein as that used for immunization. The production of PfMSP1_19_ protein (QKNG allele) has been previously described (Goodman et al., 2010). Monobiotinylated PfRH5 protein was produced for ELISAs by transient transfection of HEK293E cells (Durocher et al., 2002). The ELISA antigen encoded the version of the PfRH5 antigen expressed in the viral-vector vaccines (which lacks the CD4 d3+4 and His6 tags present in the protein vaccine). ELISAs were performed essentially according to published methodology (Sheehy et al., 2011). The OD-based ELISA results for PfAMA1 and PfRH5 were converted to μg/ml using the results of CFCA analyses, similar to that previously described (Williams et al., 2012).

Assays of GIA were performed at the PATH-MVI GIA reference laboratory, NIAID, NIH, using purified total IgG, FVO strain *P. falciparum* parasites, and a previously published method (Miura et al., 2009). A single-lifecycle assay was performed, followed by growth quantification by colorimetric detection of parasite lactate dehydrogenase. For each sample achieving >50% GIA at 2.5 mg/ml, total IgG GIA EC_50_ was calculated in terms of total IgG concentration in the well by linear interpolation. The total IgG concentration in each plasma sample was measured using Protein A biosensors on a Fortebio Blitz instrument (ForteBio). For each animal achieving >50% GIA at 2.5 mg/ml, the GIA_50_ titer was then calculated by dividing the plasma total IgG concentration by the total IgG GIA EC_50_.

### Analyses and Statistics

Throughout, all reported p values are for two-tailed tests. Vaccine efficacy endpoints were recorded, as used in a previous *Aotus*-*P. falciparum* challenge study (Lyon et al., 2008) and a study of *P. knowlesi* infection of rhesus macaques (Mahdi Abdel Hamid et al., 2011). Kendall’s tau-b was used to test a null hypothesis of equivalent outcome between Group B and historical Freund’s control animals (see Table S1 and Supplemental Information) using the ordinally ranked outcome data. As a secondary efficacy outcome measure for this group (using non-adjuvant-matched control data from the current study), LCP was compared between Groups B and A by Mann-Whitney test. The protocol-specified primary analysis of efficacy in Groups C, D, and E was comparison of LCP in each group to Group A by Mann-Whitney test with Bonferroni correction for multiple comparison. A post hoc secondary analysis of efficacy in terms of effect upon time to treatment was performed using a Mann-Whitney test with Bonferroni correction for multiple comparison, comparing each of Groups B, C, D, and E to Group A. The majority of immunological parameters were nonnormally distributed, and thus, unless detailed otherwise in the Supplemental Information, analyses of association between immunological parameters and continuous outcome variables were performed by Spearman’s rank correlation. The protocol-specified primary analysis for a correlate of protection, in the event that GIA EC_50_ data could not be estimated for every animal (as was the case here for a number of the animals in Groups C and E), was examination of the correlation between GIA at a fixed total IgG concentration and IVIG.

## Author Contributions

A.D.D., G.C.B., K.M., C.A.L., K.A.E., Y.W., G.J.W., A.G.L., and S.J.D. designed and reviewed the study and interpreted the data; A.D.D., G.C.B., J.A.V., and A.J.S. performed the cellular immunogenicity assays; A.D.D., A.D., K.M., K.H.L., K.H.M., K.A.H., C.A.L., and S.J.D. performed the humoral immunogenicity assays; A.D.D., C.C., S.J.B., J.J.I., D.G.W.A., A.V.T., Y.W., G.J.W., and S.J.D. prepared the proteins and various vaccine constructs; A.D.D., G.C.B., C.M.L., L.E.L., J.A.V., K.P.L., and Y.W. assisted with the malaria challenge and parasitological monitoring; L.A.L.-R. and J.T.M. undertook the clinical care of the *Aotus* monkeys; A.D.D. and S.J.D. performed the data and statistical analyses; and A.D.D. and S.J.D. led the study and wrote the paper with all the co-authors.

## Supplemental Information

Document S1. Supplemental Experimental Procedures and Figures S1–S5

Table S1. A Review of Previous *Aotus nancymaae*-*P. falciparum* Challenge Studies, Related to Figures 1 and S1

Document S2. Article plus Supplemental Information
