# Supplementary material for: A PfRH5-Based Vaccine Is Efficacious against Heterologous Strain Blood-Stage Plasmodium falciparum Infection in Aotus Monkeys
Source: Cell Host Microbe. 2015 Jan 14;17(1):130–9. doi: 10.1016/j.chom.2014.11.017 (PMC4297294; doi:10.1016/j.chom.2014.11.017)
Supplement: Document S2. Article plus Supplemental Information [file mmc3.pdf]

Cell Host & Microbe
Article

A PfRH5-Based Vaccine Is Efficacious against Heterologous Strain Blood-Stage *Plasmodium falciparum* Infection in *Aotus* Monkeys

Alexander D. Douglas,[1,*] G. Christian Baldeviano,[2] Carmen M. Lucas,[2] Luis A. Lugo-Roman,[2] Cécile Crosnier,[3] S. Josefin Bartholdson,[3] Ababacar Diouf,[4] Kazutoyo Miura,[4] Lynn E. Lambert,[5] Julio A. Ventocilla,[2] Karina P. Leiva,[2] Kathryn H. Milne,[1] Joseph J. Illingworth,[1] Alexandra J. Spencer,[1] Kathryn A. Hjerrild,[1] Daniel G.W. Alanine,[1] Alison V. Turner,[1] Jeromy T. Moorhead,[2] Kimberly A. Edgel,[2] Yimin Wu,[5] Carole A. Long,[4] Gavin J. Wright,[3] Andrés G. Lescano,[2] and Simon J. Draper[1]
[1]Jenner Institute, University of Oxford, Oxford OX3 7DQ, UK
[2]US Naval Medical Research Unit No. 6 (NAMRU-6), Lima, Peru
[3]Wellcome Trust Sanger Institute, Cambridge CB10 1HH, UK
[4]Laboratory of Malaria and Vector Research, NIAID/NIH, Rockville, MD 20852, USA
[5]Laboratory of Malaria Immunology and Vaccinology, NIAID/NIH, Rockville, MD 20852, USA
*Correspondence: sandy.douglas@ndm.ox.ac.uk
http://dx.doi.org/10.1016/j.chom.2014.11.017
This is an open access article under the CC BY license (http://creativecommons.org/licenses/by/3.0/).

SUMMARY

Antigenic diversity has posed a critical barrier to vaccine development against the pathogenic blood-stage infection of the human malaria parasite *Plasmodium falciparum*. To date, only strain-specific protection has been reported by trials of such vaccines in nonhuman primates. We recently showed that *P. falciparum* reticulocyte binding protein homolog 5 (PfRH5), a merozoite adhesin required for erythrocyte invasion, is highly susceptible to vaccine-inducible strain-transcending parasite-neutralizing antibody. In vivo efficacy of PfRH5-based vaccines has not previously been evaluated. Here, we demonstrate that PfRH5-based vaccines can protect *Aotus* monkeys against a virulent vaccine-heterologous *P. falciparum* challenge and show that such protection can be achieved by a human-compatible vaccine formulation. Protection was associated with anti-PfRH5 antibody concentration and in vitro parasite-neutralizing activity, supporting the use of this in vitro assay to predict the in vivo efficacy of future vaccine candidates. These data suggest that PfRH5-based vaccines have potential to achieve strain-transcending efficacy in humans.

INTRODUCTION

The development of a highly effective and deployable malaria vaccine remains an urgent priority for improving global public health. Despite recent strides in disease prevention and control, the *Plasmodium falciparum* human malaria parasite continues to exert a huge toll in terms of morbidity and mortality (Murray et al., 2012). The most advanced malaria subunit vaccine, a virus-like particle known as RTS,S, has shown only modest efficacy in young children in Phase III clinical trials (Agnandji et al., 2012), and thus new approaches are urgently needed (Moorthy et al., 2013).

RTS,S induces antibodies that reduce liver infection by the parasite (Foquet et al., 2014). An alternative and complementary strategy is to vaccinate against the subsequent blood-stage infection (which causes clinical disease and against which natural immunity is slowly acquired). Such a vaccine could prevent death and reduce incidence of disease, parasitemia, and onward transmission (Hill, 2011). However, despite 25 years of development, vaccine candidates targeting *P. falciparum*'s asexual blood stage have failed to overcome the challenge posed by the parasite's antigenic diversity. Two of the most critical road blocks have included exceptionally high thresholds for protective levels of antibody against known target antigens, coupled with problematic levels of antigen polymorphism. To date, no vaccine candidate has overcome these hurdles to achieve in vivo protection in human clinical trials (Goodman and Draper, 2010; Thera et al., 2011). In previous nonhuman primate (NHP) studies (which provide the only opportunity to study the effect of vaccines against an uninterrupted *P. falciparum* blood-stage infection), blood-stage vaccine candidates have proven protective only against vaccine-homologous parasite lines, and only when administered with non-human-compatible adjuvants (Dutta et al., 2009; Lyon et al., 2008).

P. falciparum reticulocyte-binding protein homolog 5 (PfRH5) is a recently identified merozoite protein, secreted from the apical organelles of the parasite during the red blood cell (RBC) invasion process (Baum et al., 2009). In vitro data have identified PfRH5 as the highest priority target in the blood-stage malaria vaccine field for over a decade (Douglas et al., 2011). Antibodies induced by PfRH5 vaccination of mice and rabbits overcome the two major difficulties outlined above: (i) antibodies can block erythrocyte invasion to high efficiency (with lower EC_{50} in terms of μg/ml antigen-specific antibody than against all other known antigens) (Douglas et al., 2014; Miura et al., 2009; Williams et al., 2012) and (ii) most importantly, these antibodies cross-inhibit all *P. falciparum* lines and field isolates tested to date

(Bustamante et al., 2013; Douglas et al., 2011; Reddy et al., 2014; Williams et al., 2012).

The PfRH5 protein is now known to mediate a critical non-redundant interaction with the human RBC surface protein basigin during invasion (Crosnier et al., 2011). The *PfRH5* gene is also refractory to genetic deletion (Baum et al., 2009; Hayton et al., 2008), unlike many other blood-stage antigens, confirming the essential nature of its function. In the context of natural infection, PfRH5 does not appear to be a dominant target of naturally acquired immune responses in endemic populations (Douglas et al., 2011; Tran et al., 2014; Villasis et al., 2012), but when detected, such antibody responses correlate with protective clinical outcome (Tran et al., 2014), and affinity-purified anti-PfRH5 human antibodies can neutralize parasites in vitro (Patel et al., 2013; Tran et al., 2014). The high degree of PfRH5 sequence conservation is thus associated with low-level natural immune pressure, but also functional constraints linked to basigin binding. Importantly, it has been shown that minimal amino acid substitutions in PfRH5 account for loss of basigin binding and/or host RBC tropism (linked to binding basigin orthologs from other species), suggesting the antigen may not easily escape vaccine-induced immune pressure (Hayton et al., 2008, 2013; Wanaguru et al., 2013). However, to date, no study has assessed the protective efficacy of PfRH5-based vaccines in vivo, and it remains unclear whether the encouraging observations made in vitro using an assay of parasite neutralization will translate into biologically relevant antiparasitic activity. This question is of particular importance, given the current lack of a clear correlate of vaccine efficacy against blood-stage infection in humans (Duncan et al., 2012) and the need to design improved strain-transcending malaria vaccines that can be progressed to clinical development.

In this study, we quantitatively assessed the immunogenicity of PfRH5-based vaccines delivered to *Aotus* monkeys by three different immunization regimens, including protein-in-adjuvant formulations (de Cassan et al., 2011) and an adenovirus/poxvirus vectored platform previously optimized for Phase I/IIa clinical development (Draper et al., 2008; Sheehy et al., 2012). We also evaluated the protective efficacy of these vaccines against a stringent vaccine-heterologous *P. falciparum* challenge (Stowers and Miller, 2001). This study enabled us to monitor the ability of PfRH5-based vaccines to both control and clear a virulent blood-stage infection. We report that significant protection against challenge with heterologous-strain blood-stage *P. falciparum* can be achieved in vivo by these vaccines, including when using the human-compatible viral vectored delivery platform. This protection was associated with anti-PfRH5 antibody concentration and parasite-neutralizing activity, supporting the use of this assay to predict the in vivo efficacy of future vaccine candidates. These results suggest that PfRH5-based vaccines have the potential to achieve strain-transcending efficacy in humans.

RESULTS

Evaluation of PfRH5 Vaccine Efficacy in *Aotus* Monkeys

31 *Aotus nancymaae* monkeys were randomized to groups that received protein-in-adjuvant and/or viral vectored vaccination regimes targeting either *P. falciparum* RH5 or apical membrane antigen 1 (PfAMA1), a well-studied comparator antigen that elicits strain-specific antibodies (Dutta et al., 2009; Remarque et al., 2008; Thera et al., 2011) (Figure 1A). The PfRH5 protein immunogen was pure (Figure S1A) and shown to be correctly folded by demonstration of binding to its receptor, basigin (Crosnier et al., 2011) (Figure S1B). Group A received sham vaccines, chimpanzee adenovirus serotype 63 (ChAd63) expressing *Renilla* luciferase (RLuc) prime, PBS with Abisco-100 adjuvant boost; Group B received PfRH5 protein with complete or incomplete Freund's adjuvant (CFA, IFA); Group C received ChAd63 expressing PfRH5 prime, PfRH5 protein with Abisco-100 boost; Group D received ChAd63-PfRH5 prime, modified vaccinia virus Ankara (MVA) expressing PfRH5 boost; and Group E received ChAd63-PfAMA1 prime, PfAMA1 protein with Abisco-100 boost. The ChAd63-MVA vaccine delivery platform used here has now been progressed to human clinical testing for a wide variety of difficult disease targets, including malaria, HIV-1, and hepatitis C virus (de Cassan and Draper, 2013; Draper and Heeney, 2010), while the use of mixed-modality adenoviral priming-protein-boost regimens has shown promise in small animals as well as initial clinical studies (de Cassan et al., 2011; Draper et al., 2010) (Hodgson et al., 2014). In the case of this study, the PfRH5 vaccines encoded the 3D7 allele of the antigen, while for PfAMA1 the ChAd63 vector expressed two alleles of the antigen (3D7 and FVO), and FVO allele PfAMA1 protein was used for the boost. The Group A sham-vaccinated animals served as protocol-specified infectivity controls in order to confirm consistent infection by the FVO parasite inoculum and its appropriate adaptation to growth in *Aotus*.

To evaluate the protective efficacy of the vaccines, animals were challenged 15 days after the final vaccination by intravenous administration of 10^4 PfRH5-vaccine-heterologous FVO strain *P. falciparum* infected red blood cells (iRBC) taken from a donor monkey. The parasitemia (Figures 1B–1F) and hematocrit (Hct) (Figures S1C–S1G) in the challenged animals were monitored over time. Challenge infection with this parasite line has proven highly virulent in *Aotus nancymaae* over the course of numerous studies, requiring treatment in all control animals administered complete Freund's adjuvant without a blood-stage vaccine antigen (n = 55, Table S1 and Supplemental Information). In contrast, none of the animals immunized here with PfRH5 protein in Freund's adjuvant (Group B) required treatment. Efficacy in this group was significant, both comparing treatment status versus adjuvant-matched historical controls (the protocol-specified primary analysis for this group; Kendall's $\tau_B = 0.703$, $p < 0.001$ versus historical controls) and comparing $\log_{10}$ cumulative parasitemia (LCP) up to the first day on which an animal was treated (day 10) versus Group A in the current study ($p = 0.002$ by Mann-Whitney test, Figure 1G). No parasites were seen by thin-film microscopy at any point in two of the animals, with only a single parasite seen on one occasion in a third animal; the remaining three animals self-cured after periods of microscopically patent parasitemia at levels <10,000 parasites/μl (p/μl) (Figure 1C). To our knowledge, such robust protection has not been observed even after vaccine-homologous challenge of *Aotus* immunized with *P. falciparum* AMA1, merozoite surface protein 1 (PfMSP1), PfMSP3, or the erythrocyte binding antigen 175 kDa (PfEBA175) formulated with Freund's adjuvant (Hisaeda et al., 2002; Jones et al., 2001; Stowers et al., 2001, 2002). Here, the PfRH5 antigen of the challenge strain, FVO,

A

Group	Day 0	Day 26	Day 54	*n* =
A	ChAd63-RLuc	–	PBS – Abisco-100	6
B	PfRH5 – CFA	PfRH5 – IFA	PfRH5 – IFA	6
C	ChAd63-PfRH5	–	PfRH5 – Abisco-100	7
D	ChAd63-PfRH5	–	MVA-PfRH5	6
E	ChAd63-PfAMA1	–	PfAMA1 – Abisco-100	6

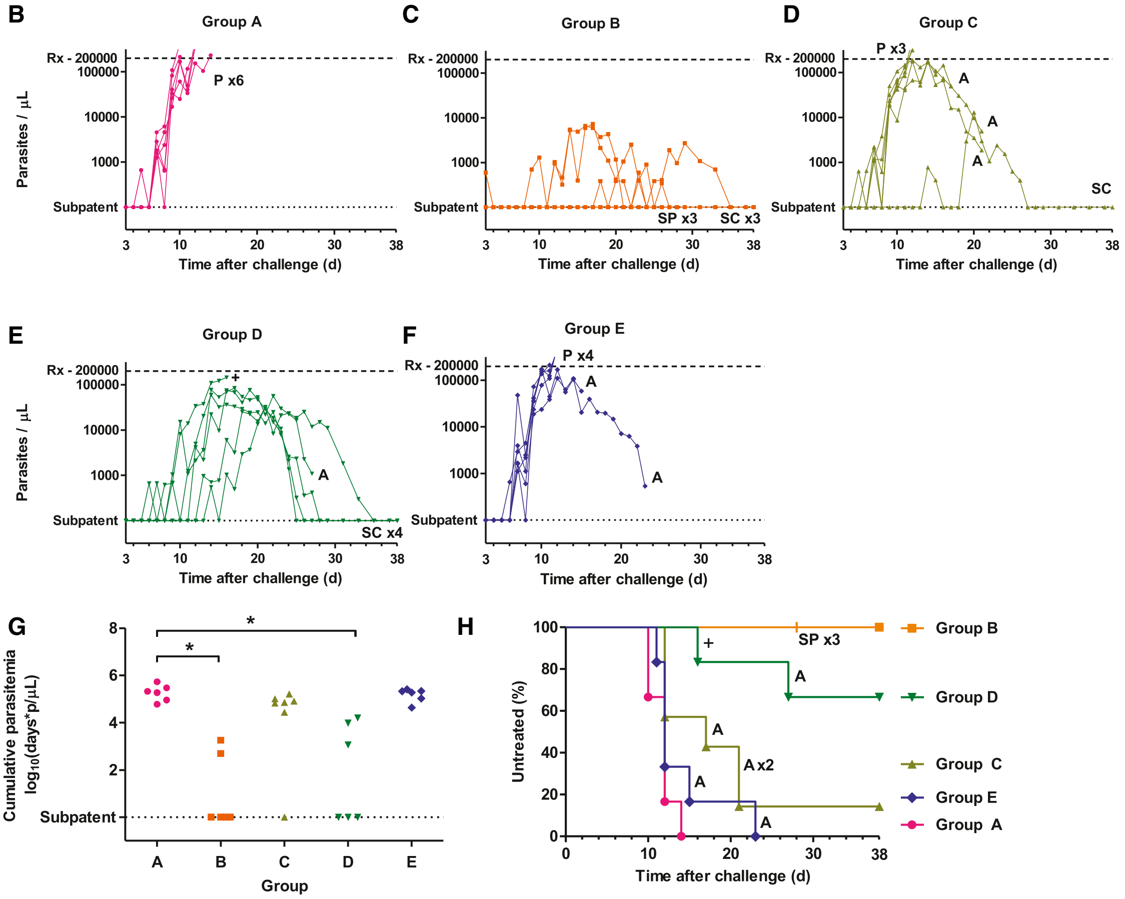

Figure 1. PfRH5 Vaccines Protect against *P. falciparum* Challenge

(A) Vaccination regimes. Immunizations were performed by the intramuscular route on days 0 and 54, with the exception of those containing Freund's adjuvant, which were given subcutaneously on days 0, 26, and 54. Doses used were 5×10^9 infectious units (ifu) for ChAd63 vaccines, 2×10^8 plaque-forming units (pfu) for MVA, 50 µg for protein vaccines, 250 µl for Freund's adjuvant, and 48 µg for Abisco-100.

(B–F) Time course of parasitemia for individual animals in Group A (B), Group B (C), Group C (D), Group D (E), and Group E (F). Upper horizontal dashed line indicates the 200,000 p/µl threshold for initiation of antimalarial treatment (Rx) because of hyperparasitemia; lower horizontal dotted line indicates absence of thin-film detectable parasites. P indicates treatment due to hyperparasitemia; A indicates treatment due to anemia; SPx3′ at day 28 in Group B indicates cessation of follow-up of three animals that had been microscopically subpatent since day 4; SC indicates self-cure in animals that had experienced sustained parasitemia; + indicates a single animal found dead on day 16; occasional unexpected deaths have previously been recorded among *Aotus* both before and during *P. falciparum* challenge (Darko et al., 2005; Hisaeda et al., 2002; Singh et al., 2006).

(G) Cumulative parasitemia up to day 10, the first day on which an animal was treated. *p < 0.01 versus Group A by Mann-Whitney test performed with Bonferroni correction for multiple comparison (this was the prespecified primary analysis for Groups C–E; secondary analysis for Group B).

(H) Kaplan-Meier plot of percentage untreated survival by group. Symbols are as in (B)–(F). Comparing time to treatment in each group to Group A was done by Mann-Whitney test with Bonferroni correction for multiple comparison, p = 0.02 for each of Groups B and D.

See also Figures S1 and S5 and Table S1.

A

B

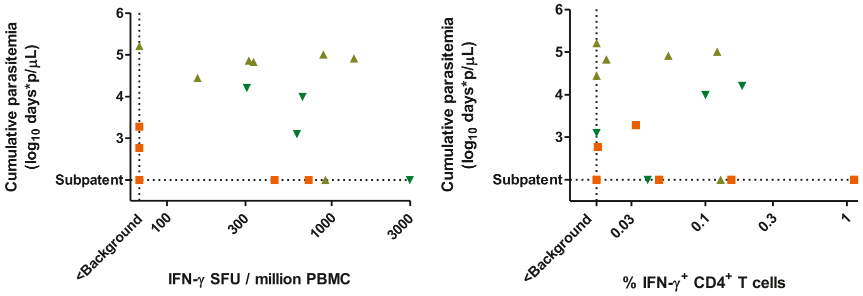

C

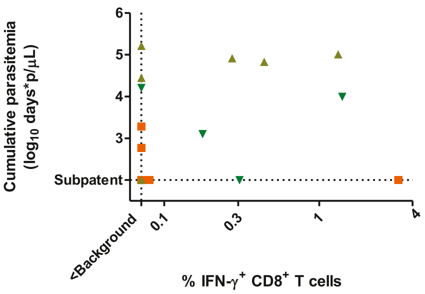

D

	LCP		Peak parasitemia	
	r_s	P	r_s	P
ELISpot	-0.04	0.89	0.34	0.18
ICS CD4$^+$	-0.23	0.39	-0.13	0.64
ICS CD8$^+$	0.10	0.71	0.23	0.38

Figure 2. Lack of Relationship between ELISpot and ICS Responses and Challenge Outcome

(A–C) Relationships between challenge outcome (LCP) and PfRH5-specific ELISpot responses (A), IFN-γ$^+$ CD4$^+$ T cell responses (B), and IFN-γ$^+$ CD8$^+$ T cell responses (C) in PfRH5-vaccinated animals (n = 17 for ELISpot, n = 16 for ICS; Groups B, C, D, excluding the two animals for which ELISpot results were not obtained and the three animals for which ICS results were not obtained). All panels plot responses after subtraction of responses in negative control unstimulated wells. Dashed line marked "< Background" indicates responses of less than the mean plus three standard deviations (SD) of the apparent response in Group A animals.

(D) Lack of statistically significant Spearman's rank correlation between ELISpot and ICS-measured immunological parameters and outcome parameters (LCP or peak parasitemia; no Bonferroni correction was made). Measurements that did not exceed background (defined as above) were treated as tied observations.

See also Figure S2.

differed at four amino acid loci from the 3D7 clone upon which the vaccine was based (Hayton et al., 2008). No greater level of divergence from the 3D7 antigen was identified among 227 field parasite strains recently sequenced (Manske et al., 2012; Williams et al., 2012).

Significant vaccine efficacy was also observed in the animals immunized with human-compatible ChAd63-MVA PfRH5 vaccines (Group D), as judged using the prespecified primary endpoint for Groups C–E of LCP up to day 10 as compared to Group A (Figure 1G; p = 0.007 by Mann-Whitney test with Bonferroni correction for multiple comparison). Similar to Freund's adjuvant, there are abundant pre-existing data across multiple species to demonstrate that there is no nonspecific effect of the viral vector vaccines used in Group D upon the rate of *Plasmodium* blood-stage infection, including for *P. falciparum* in malaria-challenged humans as compared to unvaccinated infectivity controls (Ewer et al., 2013; Sheehy et al., 2012), as well as in three rodent malaria species: *P. yoelii*, *P. berghei*, or *P. chabaudi* (Biswas et al., 2012; Goodman et al., 2013). There was also no nonspecific effect of vaccination with COPAK (a poxvirus similar to the MVA) upon the course of *P. knowlesi* parasitemia in rhesus macaques (Weiss et al., 2007). However, as is typical in this model, a number of animals that developed but controlled relatively high-level parasitemia in Groups C and D subsequently required treatment due to anemia (Figures 1H and S1C–S1G), the severity and timing of which correlated with the level and timing of peak parasitemia (Figures S2H–S2J). Most importantly, unlike previous trials of a human-compatible blood-stage vaccine delivery platform in this model, self-cure of infection without severe anemia was observed in 4/6 animals in the ChAd63-MVA PfRH5 group and 1/7 in the ChAd63-Protein PfRH5 group (Figures 1D, 1E, and 1H). The four self-curing animals in the ChAd63-MVA PfRH5 group experienced median peak parasite density of 48,000 p/μl (median peak percentage parasitemia 0.9%) and were afebrile and clinically well throughout.

Immunologic Correlates of Protection

The rationale for the development of these PfRH5 vaccines was the observation that PfRH5-specific antibodies could achieve high levels of parasite-neutralizing in vitro growth inhibitory activity (GIA) (Bustamante et al., 2013; Douglas et al., 2011; Williams et al., 2012). We therefore hypothesized prior to the trial that protection achieved by PfRH5 vaccines would be associated with anti-PfRH5 antibody concentrations and in vitro GIA, but not necessarily with cellular responses against PfRH5. To test this hypothesis, we initially assessed humoral and cellular immune responses by ELISA, interferon-γ (IFN-γ) ELISpot, and intracellular cytokine staining (ICS) assays.

Antigen-specific T cell responses to a subunit vaccine have rarely been measured in *Aotus*, but we were able to detect PfRH5 and PfAMA1-specific IFN-γ-producing PBMCs by ELISpot (Figures S2A–S2C) and resolve these into CD4$^+$ and CD8$^+$ T cells by ICS (Figures S2D–S2G). There was no correlation between ELISpot or ICS responses and LCP or peak parasitemia (Figures 2A–2D).

The vaccines also induced substantial PfRH5-specific antibody responses, as measured by ELISA with conversion into absolute antigen-specific antibody concentrations achieved via surface plasmon resonance (SPR) calibration-free concentration analysis (CFCA) (Williams et al., 2012) (Figures 3A, S3, and S4A–S4F). Geometric mean day-of-challenge (DoC) PfRH5-specific antibody concentrations achieved were 700 μg/ml in Group B (PfRH5 protein in Freund's adjuvant), 54 μg/ml in Group C (ChAd63-Protein PfRH5), and 320 μg/ml in Group D (ChAd63-MVA PfRH5); the corresponding geometric mean anti-PfAMA1 (FVO allele)-specific antibody concentration in Group E was 140 μg/ml. There was a strong and statistically significant

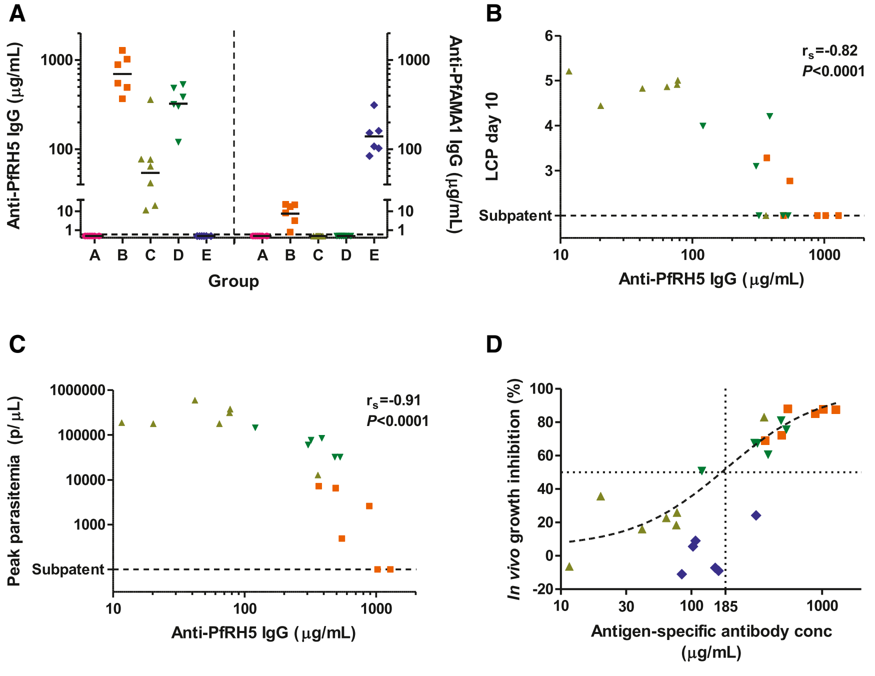

Figure 3. Protection Is Associated with Prechallenge Anti-PfRH5 Antibody Concentration

(A) Plasma were tested by ELISA on the day of challenge (DoC, day 69) for anti-PfRH5 (3D7) allele (left y axis) or anti-PfAMA1 (FVO) allele (right y axis) total IgG responses. Individual responses and geometric mean are shown for each group. Arbitrary ELISA units were converted to μg/ml concentrations following definition of a conversion factor by CFCA (Figure S3).

(B–D) Relationships between DoC plasma antibody concentration and challenge outcome. For the 19 PfRH5-vaccinated animals, Spearman's rank correlation coefficient (r_s) and p value are shown for the relationship of anti-PfRH5 total IgG concentration on DoC with the primary endpoint (LCP) (B) and peak parasitemia (C). (D) The relationship between IVGI and antigen-specific antibody concentration (anti-PfRH5 in the 19 PfRH5 vaccinated animals, and anti-PfAMA1 for the 6 PfAMA1-vaccinated animals). A nonlinear regression curve was fitted to the points from the PfRH5-vaccinated animals and used to estimate IVGI EC_{50}, as reported in the text. There were insufficient data for curve fitting to the PfAMA1-vaccinated animals.

See also Figures S3 and S4.

correlation between anti-PfRH5 antibody concentration and challenge outcome (Figures 3B–3D). These correlations were maintained when the analysis was conducted among animals *within* Groups B and D (Figures S4G and S4H). Given that these animals serve as matched controls for each other within a group, such a correlation would be unlikely if protection was attributable to a nonspecific effect of the adjuvant or the viral vaccine vector.

In vivo growth inhibition was also calculated (IVGI; the percentage reduction in the parasite multiplication rate (PMR) in each animal relative to the mean in the control group, as has been previously described; Mahdi Abdel Hamid et al., 2011). Using nonlinear least-squares regression, the concentration of anti-PfRH5 immunoglobulin G (IgG) required to achieve 50% IVGI (IVGI EC_{50}) was estimated at 185 μg/ml (95% CI 100–330 μg/ml, using data from Groups B–D). The relationship between PfAMA1-specific antibody concentration and IVGI was suggestive of a higher IVGI EC_{50} for anti-PfAMA1 IgG than for anti-PfRH5 IgG (Figure 3D). These in vivo growth inhibition data are thus in line with previous in vitro GIA data using purified antigen-specific anti-PfRH5 and anti-PfAMA1 IgG from rabbits and humans (Miura et al., 2009; Tran et al., 2014), suggesting that parasites are quantitatively more susceptible to anti-PfRH5 than to anti-PfAMA1 antibodies.

We subsequently assessed the functional ability of the vaccine-induced antibody responses to neutralize parasites in the GIA assay. The ability of a range of concentrations of protein G-purified total IgG to neutralize FVO (challenge-strain) parasites was assessed (Figure 4A). There was a strong and statistically significant relationship between GIA at 2.5 mg/ml total IgG and IVGI, the protocol-specified primary analysis for an immunological correlate of protection (Figure 4B). Immunological correlates of vaccine-induced protection against other pathogens are commonly the attainment of a particular level of in vitro activity at a certain serum dilution titer (Plotkin, 2010). Here, we observed that the total plasma IgG concentration of animals immunized with different regimes differed substantially (Figure 4C). We therefore calculated a GIA_{50} titer, defined as the dilution factor from the plasma IgG concentration to the IgG concentration achieving 50% GIA. GIA_{50} titer was closely associated with challenge outcome (Figure 4D), with attainment of a GIA_{50} titer exceeding five appearing predictive of untreated survival.

Impact of Infection on Vaccine-Induced Responses

We also monitored immune responses in the period following the challenge infection. Consistent with the poor immunogenicity of PfRH5 in the context of natural infection (Douglas et al., 2011; Tran et al., 2014; Villasis et al., 2012), the effect of parasite exposure on PfRH5-specific antibody levels was variable (Figure 5A) when assessing the day of challenge (day 69) versus the end of the challenge follow-up period (day 107). In contrast, median anti-PfAMA1 IgG levels increased across all the groups, but most notably in Group E (Figure 5B). Nearly every animal experiencing a patent infection seroconverted to the 19 kDa C terminus of PfMSP1 ($PfMSP1_{19}$; Figure 5C), consistent with the abundant and immunodominant nature of this merozoite surface antigen, supported by similar data from controlled human malaria infection (CHMI) studies in malaria-naive adult volunteers (Elias et al., 2014). Changes in GIA measured at a constant total IgG concentration of 2.5 mg/ml in the same period were small, with no significant change in PfRH5-vaccinated, PfAMA1-vaccinated, or sham-vaccinated animals (median changes of −7%, −3%, and 6%; p = 0.21, 0.81, and 0.14, respectively, by Wilcoxon signed-rank test). Nonetheless, GIA_{50} titers increased substantially in many cases, due to increases in total plasma IgG concentration after parasite exposure (Figures 5D and 5E). Follow-up of animals to day 230 (161 days after challenge)

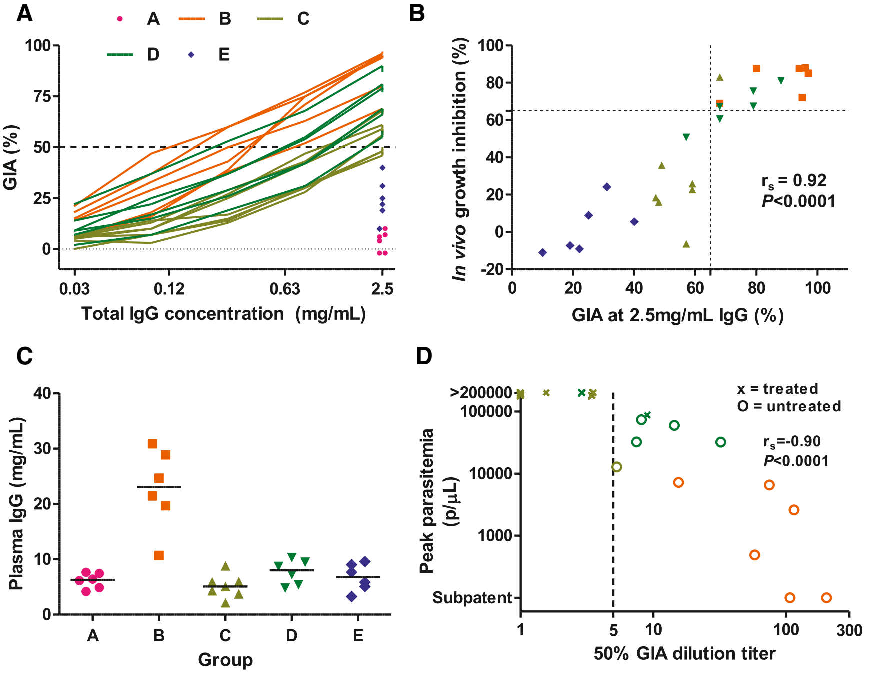

Figure 4. Attainment of 50% GIA at a Sub-physiological IgG Concentration Predicts Protection

(A) The in vitro GIA of purified IgG (DoC time point) was assessed against FVO strain parasites for all animals at a fixed concentration of 2.5 mg/ml. Percent GIA is reported following a single cycle of parasite growth. For animals in Groups B, C, and D, the assays were repeated with a dilution series of the purified IgG. The results for each individual animal are shown.

(B) Prespecified primary analysis for an immunological correlate of protection: relationship between percent GIA using 2.5 mg/ml purified IgG in the assay and the percent IVGI modeled from the parasitemia data. Across all animals (n = 31), Spearman's r_s = 0.86, p < 0.0001; among non-control vaccinated animals only (Groups B–E, n = 25, as shown), r_s = 0.92, p < 0.0001. The dashed lines identify animals in the top right quadrant that did not require treatment following challenge. GIA was predictive of outcome independent of group allocation (p = 0.004 by likelihood-ratio test comparing a bivariate model relating IVGI with group versus a multivariate model relating IVGI with group plus GIA at 2.5 mg/ml).

(C) The plasma concentration of IgG in mg/ml was assayed at the DoC time point. Individual results and group medians are reported.

(D) GIA_{50} titer was calculated for Groups B, C, and D (by dividing the plasma total IgG concentration by the total IgG GIA EC_{50}) and is plotted against peak parasitemia. Three animals in Group C, which all required treatment, did not achieve 50% GIA at 2.5 mg/ml and were assigned tied GIA_{50} titers of 1. Spearman's rank correlation coefficient is shown.

See also Figure S5.

demonstrated that, in several animals, anti-PfRH5 antibody concentrations were maintained at levels in excess of the IVGI EC_{50} for a number of months after challenge (Figure S5A).

DISCUSSION

Overall, these results demonstrate that PfRH5-based vaccines have the potential to overcome the shortcomings of previous blood-stage vaccines against *P. falciparum* (Wright and Rayner, 2014). The attainment of cross-strain protection in vivo by an antigen selected on the basis of its ability to induce cross-strain GIA in vitro strongly hints that the relationship between GIA and protection is causal, encouraging the continued use of the assay for candidate vaccine selection. Our findings are also consistent with previous data linking protection with the attainment of 60% GIA against the challenge strain at 2.5 mg/ml total IgG, suggesting that the quantitative relationship between GIA and protection may be roughly similar for different antigens (Singh et al., 2006). Importantly, the efficacy observed here would also have been readily detectable in a Phase IIa CHMI clinical trial (Figures S5B and S5C). These data thus support the assertion that an efficacious blood-stage vaccine candidate should be able to demonstrate in vivo biological effects in CHMI trials prior to field trials (Sheehy et al., 2013).

Protection in this study appeared to be mediated by preformed anti-PfRH5 antibody present at the time of infection, as distinct from a recall response against PfRH5 after challenge. Antigen-specific antibody concentrations exceeding 100 μg/ml have been attained in humans by other malaria vaccines including RTS,S and those targeting PfAMA1 (Kester et al., 2009; Spring et al., 2009), but long-term maintenance of such high-level responses may be challenging, particularly if *P. falciparum* infection does not appreciably boost vaccine-induced anti-PfRH5 responses. We have recently demonstrated that antibodies of other specificities can act synergistically with anti-PfRH5 antibodies in GIA assays, thus supporting an ongoing strategy to achieve protection with substantially lower and more easily maintained antibody concentrations (Williams et al., 2012).

Like the functionally critical surface proteins of other challenging vaccine targets, such as HIV-1 and influenza virus, the immunodominant antigens of *Plasmodium* spp. are highly variable (Riley and Stewart, 2013). PfRH5 is also functionally critical, but analogous to the pre-erythrocytic malaria antigen circumsporozoite protein (the basis of the RTS,S vaccine), the response to it in the context of infection is unlikely to be of sufficient magnitude to be a substantial contributor to natural immunity (Douglas et al., 2011; Murungi et al., 2013; Tran et al., 2014). Although this immune evasion strategy is clearly successful in permitting *P. falciparum* to establish repeated and chronic infections, it has left a conserved whole-protein target that appears more susceptible to subunit vaccination than the conserved epitopes presented by HIV-1 and influenza.

The clinical implications of these data for PfRH5-based vaccines remain unknown for now and will require clinical trials to assess immunogenicity and efficacy in humans (Figure S5D). However, given the attainment of an unprecedented level of protection in a stringent model, this study has clearly demonstrated

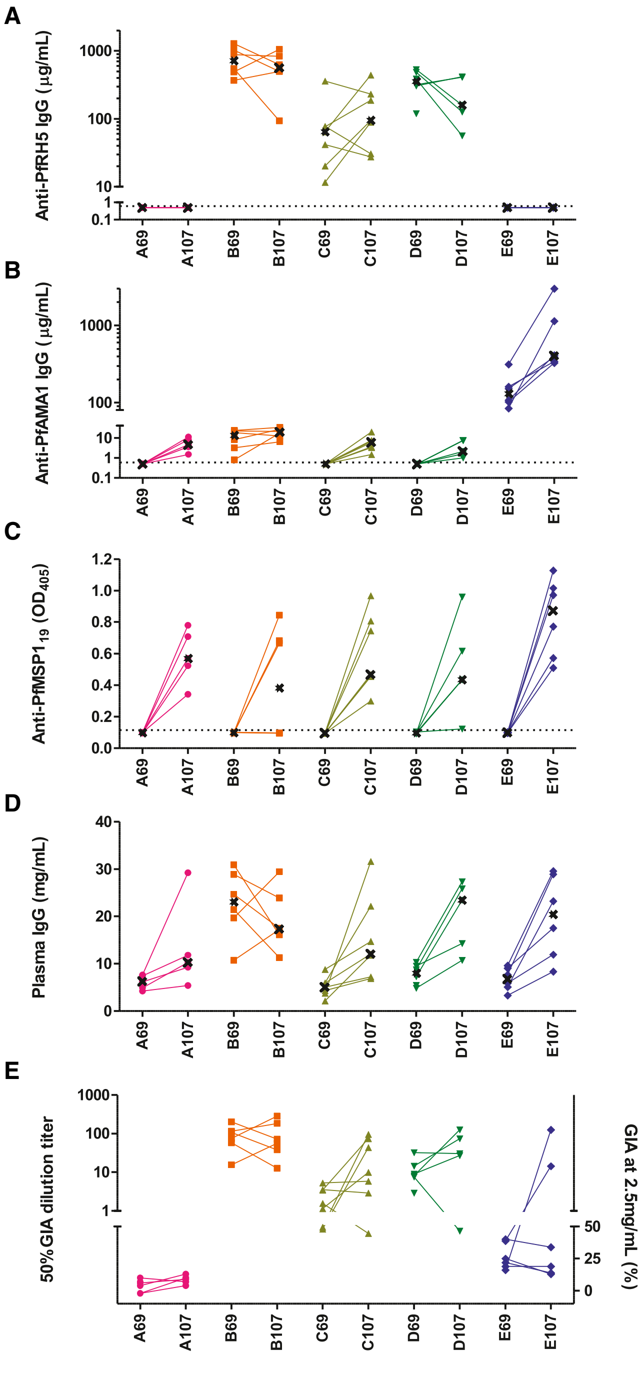

Figure 5. Post-Challenge Measures of Immunogenicity

(A–E) Plasma antigen-specific total IgG responses and GIA were also assessed in the post-challenge period. Individual responses are shown and linked for each animal in each group at the DoC time point (day 69) and at the end of the challenge monitoring period (day 107, 38 days post-challenge). Median responses are indicated, where possible, by a black cross. Anti-PfRH5 (A), anti-PfAMA1 (B), and anti-PfMSP1$_{19}$ (C) ELISA data are shown. (D) Plasma IgG concentration in mg/ml. (E) GIA was assessed as in Figure 4, initially testing all samples at 2.5 mg/ml purified IgG. If >50% GIA was observed, samples were diluted in the assay, and the GIA$_{50}$ titer was calculated. For each animal at each time point, GIA$_{50}$ titer is shown on the top left y axis (where possible to calculate); otherwise, the percent GIA at 2.5 mg/ml is indicated on the lower right y axis.
See also Figure S5.

that the problem of interstrain blood-stage antigen variation is tractable. It is worth noting that the FVO strain of *P. falciparum* has been selected for rapid growth and virulence in *Aotus* monkeys; as a result, it is considerably more virulent in these animals (essentially universally fatal in untreated animals) than is the case for *P. falciparum* in humans (in which the probability of severe disease is thought not to exceed 15% per episode even in malaria-naive infants) (Collins et al., 1994; Gupta et al., 1999). Moreover, having defined two significant correlates of protection, it will now be possible to rationally improve PfRH5-based immunogens and their delivery in order to maximize functional antiparasitic immunity. The optimization of PfRH5 vaccine formulations to achieve and maintain the highest possible levels of antibody will also be a major focus of human clinical trials that should commence within the coming year, supported by a greater weight of preclinical evidence than any previous blood-stage vaccine candidate. In summary, this study provides the initial proof of concept in NHPs that the development of a strain-transcending blood-stage vaccine against *P. falciparum* is possible and provides important insight into a correlate of protection against the human malaria parasite.

EXPERIMENTAL PROCEDURES

Full experimental methods are provided in detail in the Supplemental Information.

Vaccines

All PfRH5 vaccines were based upon the *P. falciparum* 3D7 clone sequence. The production of recombinant MVA expressing full-length PfRH5 has previously been described (Douglas et al., 2011). For the current study, a ChAd63 vector expressing the same PfRH5 transgene was produced using previously described methods (Goodman et al., 2010). The production of the ChAd63 and protein PfAMA1 (FVO allele) vaccines have been reported elsewhere (Biswas et al., 2011; Kennedy et al., 2002). The PfRH5 3D7 protein was expressed and tested for basigin binding essentially as described (Crosnier et al., 2011).

Animals, Immunizations, Challenge, and Sample Collection

Adult female owl monkeys (*Aotus nancymaae*) were housed at the US Naval Medical Research Unit No. 6 (NAMRU-6). Randomization to groups was stratified by pretrial weight. All immunizations were administered under ketamine anesthesia and performed by the intramuscular route (into the caudal quadriceps), with the exception of those containing Freund's adjuvant, which were given subcutaneously (into the interscapular area). The study protocol was approved by NAMRU-6's Institutional Animal Care and Use Committee (protocol number NAMRU-6 11-12), the Department of the Navy Bureau of Medicine and Surgery (NRD-748), the University of Oxford Animal Care and Ethical Review Committee, and the Institut Nacional de Recursos Naturales (INRENA) at the Peruvian Ministry of Agriculture.

15 days after the final vaccination, animals were challenged intravenously with 10^4 FVO-strain *P. falciparum* iRBC taken from a donor monkey, as previously described (Stowers et al., 2001). From day 72, daily thin-film parasitemia quantification and alternate-day Hct measurements were conducted. Animals were treated (i) when parasite density reached $\geq$200,000/μl, (ii) when Hct fell to $\leq$25%, (iii) upon reaching challenge day 28 (C+28) if no parasites had been seen in the preceding week, or (iv) upon reaching C+38 (study day 107). Blood samples for immunological assays were collected from all animals' saphenous veins under ketamine anesthesia. EDTA-anticoagulated blood was prepared using standard methods to obtain plasma and PBMC.

Cellular Immune Assays

Ex vivo IFN-γ ELISpot and ICS were performed essentially as previously described (Draper et al., 2010). Assays used frozen PBMC and pools of PfRH5 and PfAMA1 peptides.

ELISA, CFCA, and GIA

PfAMA1 ELISAs used the same recombinant PfAMA1 FVO protein as that used for immunization. The production of $PfMSP1_{19}$ protein (QKNG allele) has been previously described (Goodman et al., 2010). Monobiotinylated PfRH5 protein was produced for ELISAs by transient transfection of HEK293E cells (Durocher et al., 2002). The ELISA antigen encoded the version of the PfRH5 antigen expressed in the viral-vector vaccines (which lacks the CD4 d3+4 and His6 tags present in the protein vaccine). ELISAs were performed essentially according to published methodology (Sheehy et al., 2011). The OD-based ELISA results for PfAMA1 and PfRH5 were converted to μg/ml using the results of CFCA analyses, similar to that previously described (Williams et al., 2012).

Assays of GIA were performed at the PATH-MVI GIA reference laboratory, NIAID, NIH, using purified total IgG, FVO strain *P. falciparum* parasites, and a previously published method (Miura et al., 2009). A single-lifecycle assay was performed, followed by growth quantification by colorimetric detection of parasite lactate dehydrogenase. For each sample achieving >50% GIA at 2.5 mg/ml, total IgG GIA EC_{50} was calculated in terms of total IgG concentration in the well by linear interpolation. The total IgG concentration in each plasma sample was measured using Protein A biosensors on a Fortebio Blitz instrument (ForteBio). For each animal achieving >50% GIA at 2.5 mg/ml, the GIA_{50} titer was then calculated by dividing the plasma total IgG concentration by the total IgG GIA EC_{50}.

Analyses and Statistics

Throughout, all reported p values are for two-tailed tests. Vaccine efficacy endpoints were recorded, as used in a previous *Aotus-P. falciparum* challenge study (Lyon et al., 2008) and a study of *P. knowlesi* infection of rhesus macaques (Mahdi Abdel Hamid et al., 2011). Kendall's tau-b was used to test a null hypothesis of equivalent outcome between Group B and historical Freund's control animals (see Table S1 and Supplemental Information) using the ordinally ranked outcome data. As a secondary efficacy outcome measure for this group (using non-adjuvant-matched control data from the current study), LCP was compared between Groups B and A by Mann-Whitney test. The protocol-specified primary analysis of efficacy in Groups C, D, and E was comparison of LCP in each group to Group A by Mann-Whitney test with Bonferroni correction for multiple comparison. A post hoc secondary analysis of efficacy in terms of effect upon time to treatment was performed using a Mann-Whitney test with Bonferroni correction for multiple comparison, comparing each of Groups B, C, D, and E to Group A. The majority of immunological parameters were nonnormally distributed, and thus, unless detailed otherwise in the Supplemental Information, analyses of association between immunological parameters and continuous outcome variables were performed by Spearman's rank correlation. The protocol-specified primary analysis for a correlate of protection, in the event that GIA EC_{50} data could not be estimated for every animal (as was the case here for a number of the animals in Groups C and E), was examination of the correlation between GIA at a fixed total IgG concentration and IVIG.

SUPPLEMENTAL INFORMATION

Supplemental Information includes Supplemental Experimental Procedures, five figures, and one table and can be found with this article online at http://dx.doi.org/10.1016/j.chom.2014.11.017.

AUTHOR CONTRIBUTIONS

A.D.D., G.C.B., K.M., C.A.L., K.A.E., Y.W., G.J.W., A.G.L., and S.J.D. designed and reviewed the study and interpreted the data; A.D.D., G.C.B., J.A.V., and A.J.S. performed the cellular immunogenicity assays; A.D.D., A.D., K.M., K.H.L., K.H.M., K.A.H., C.A.L., and S.J.D. performed the humoral immunogenicity assays; A.D.D., C.C., S.J.B., J.J.I., D.G.W.A., A.V.T., Y.W., G.J.W., and S.J.D. prepared the proteins and various vaccine constructs; A.D.D., G.C.B., C.M.L., L.E.L., J.A.V., K.P.L., and Y.W. assisted with the malaria challenge and parasitological monitoring; L.A.L.-R. and J.T.M. undertook the clinical care of the *Aotus* monkeys; A.D.D. and S.J.D. performed the data and statistical analyses; and A.D.D. and S.J.D. led the study and wrote the paper with all the co-authors.

ACKNOWLEDGMENTS

The authors are grateful for the assistance of Adrian Hill, Julie Furze, the Viral Vector Core Facility and Adjuvant Bank (Jenner Institute, University of Oxford); David Staunton (Biophysical Instrument Facility, Department of Biochemistry, University of Oxford); Ly-Mee Yu and Doug Altman (Centre for Statistics in Medicine, University of Oxford); Julian Rayner (Wellcome Trust Sanger Institute, UK); Carmen Franco, Roxana Lescano, Jorge Nuñez, Meddly Santolalla, and Lorena Tapia (NAMRU-6, Peru); Olivo Miotto (Mahidol-Oxford Research Unit, Thailand); Yves Durocher for provision of HEK293E cells (CNRC-NRC, Canada); and Alfredo Nicosia (Okairòs, Italy) for provision of the ChAd63 vector. A.D.D. held a Wellcome Trust Training Fellowship for Clinicians in Basic Sciences (grant number 089455/2/09/z). J.J.I. is a Wellcome Trust funded student on the Infection, Immunology and Translational Medicine PhD Programme (grant number 092873/z/10/z). C.C., S.J.B., and G.J.W. are supported by the Wellcome Trust (grant number 098051). The GIA work was supported by the PATH Malaria Vaccine Initiative and the Intramural Program of the National Institutes of Health, National Institute of Allergy and Infectious Diseases. This work was also funded in part by the University Challenge Seed Fund (Isis Innovation, University of Oxford) and by the European Community's Seventh Framework Programme (FP7/2007-2013) under grant agreement number 242095 – EVIMalaR. A.G.L. is supported by training grant NIH/FIC 2D43 TW007393 awarded to NAMRU-6 by the Fogarty International Center of the US National Institutes of Health. S.J.D. holds a MRC Career Development Fellowship (grant number G1000527; this fellowship is jointly funded by the UK Medical Research Council [MRC] and the UK Department for International Development [DFID] under the MRC/DFID Concordat agreement) and is a Jenner Investigator and Lister Institute Research Prize Fellow. A.D.D., J.J.I., C.C., S.J.B., G.J.W., and S.J.D. are named on patent applications relating to PfRH5 and/or other malaria vaccines. Some of the listed authors are either military service members (K.A.E., L.A.L.-R., J.T.M.) or employees of the US Government (G.C.B., C.M.L., J.A.V., K.P.L., A.G.L.). This work was prepared as part of their official duties. Title 17 U.S.C. §105 provides that "Copyright protection under this title is not available for any work of the United States Government." Title 17 U.S.C. §101 defines a U.S. Government work as a work prepared by a military service member or employee of the US Government as part of that person's official duties. The views expressed in this article are those of the authors and do not necessarily reflect the official policy or position of the Department of the Navy, Department of Defense, or the US Government.

Received: May 17, 2014
Revised: September 11, 2014
Accepted: November 13, 2014
Published: January 14, 2015

REFERENCES

Agnandji, S.T., Lell, B., Fernandes, J.F., Abossolo, B.P., Methogo, B.G., Kabwende, A.L., Adegnika, A.A., Mordmüller, B., Issifou, S., Kremsner, P.G., et al.; RTS,S Clinical Trials Partnership (2012). A phase 3 trial of RTS,S/AS01 malaria vaccine in African infants. N. Engl. J. Med. *367*, 2284–2295.

Baum, J., Chen, L., Healer, J., Lopaticki, S., Boyle, M., Triglia, T., Ehlgen, F., Ralph, S.A., Beeson, J.G., and Cowman, A.F. (2009). Reticulocyte-binding protein homologue 5 - an essential adhesin involved in invasion of human erythrocytes by Plasmodium falciparum. Int. J. Parasitol. *39*, 371–380.

Biswas, S., Dicks, M.D., Long, C.A., Remarque, E.J., Siani, L., Colloca, S., Cottingham, M.G., Holder, A.A., Gilbert, S.C., Hill, A.V., and Draper, S.J. (2011). Transgene optimization, immunogenicity and in vitro efficacy of viral vectored vaccines expressing two alleles of Plasmodium falciparum AMA1. PLoS ONE *6*, e20977.

Biswas, S., Spencer, A.J., Forbes, E.K., Gilbert, S.C., Holder, A.A., Hill, A.V., and Draper, S.J. (2012). Recombinant viral-vectored vaccines expressing Plasmodium chabaudi AS apical membrane antigen 1: mechanisms of vaccine-induced blood-stage protection. J. Immunol. *188*, 5041–5053.

Bustamante, L.Y., Bartholdson, S.J., Crosnier, C., Campos, M.G., Wanaguru, M., Nguon, C., Kwiatkowski, D.P., Wright, G.J., and Rayner, J.C. (2013). A

full-length recombinant Plasmodium falciparum PfRH5 protein induces inhibitory antibodies that are effective across common PfRH5 genetic variants. Vaccine *31*, 373–379.

Collins, W.E., Galland, G.G., Sullivan, J.S., and Morris, C.L. (1994). Selection of different strains of Plasmodium falciparum for testing blood-stage vaccines in Aotus nancymai monkeys. Am. J. Trop. Med. Hyg. *51*, 224–232.

Crosnier, C., Bustamante, L.Y., Bartholdson, S.J., Bei, A.K., Theron, M., Uchikawa, M., Mboup, S., Ndir, O., Kwiatkowski, D.P., Duraisingh, M.T., et al. (2011). Basigin is a receptor essential for erythrocyte invasion by Plasmodium falciparum. Nature *480*, 534–537.

Darko, C.A., Angov, E., Collins, W.E., Bergmann-Leitner, E.S., Girouard, A.S., Hitt, S.L., McBride, J.S., Diggs, C.L., Holder, A.A., Long, C.A., et al. (2005). The clinical-grade 42-kilodalton fragment of merozoite surface protein 1 of Plasmodium falciparum strain FVO expressed in Escherichia coli protects Aotus nancymai against challenge with homologous erythrocytic-stage parasites. Infect. Immun. *73*, 287–297.

de Cassan, S.C., and Draper, S.J. (2013). Recent advances in antibody-inducing poxviral and adenoviral vectored vaccine delivery platforms for difficult disease targets. Expert Rev. Vaccines *12*, 365–378.

de Cassan, S.C., Forbes, E.K., Douglas, A.D., Milicic, A., Singh, B., Gupta, P., Chauhan, V.S., Chitnis, C.E., Gilbert, S.C., Hill, A.V., and Draper, S.J. (2011). The requirement for potent adjuvants to enhance the immunogenicity and protective efficacy of protein vaccines can be overcome by prior immunization with a recombinant adenovirus. J. Immunol. *187*, 2602–2616.

Douglas, A.D., Williams, A.R., Illingworth, J.J., Kamuyu, G., Biswas, S., Goodman, A.L., Wyllie, D.H., Crosnier, C., Miura, K., Wright, G.J., et al. (2011). The blood-stage malaria antigen PfRH5 is susceptible to vaccine-inducible cross-strain neutralizing antibody. Nat. Commun. *2*, 601.

Douglas, A.D., Williams, A.R., Knuepfer, E., Illingworth, J.J., Furze, J.M., Crosnier, C., Choudhary, P., Bustamante, L.Y., Zakutansky, S.E., Awuah, D.K., et al. (2014). Neutralization of Plasmodium falciparum merozoites by antibodies against PfRH5. J. Immunol. *192*, 245–258.

Draper, S.J., and Heeney, J.L. (2010). Viruses as vaccine vectors for infectious diseases and cancer. Nat. Rev. Microbiol. *8*, 62–73.

Draper, S.J., Moore, A.C., Goodman, A.L., Long, C.A., Holder, A.A., Gilbert, S.C., Hill, F., and Hill, A.V. (2008). Effective induction of high-titer antibodies by viral vector vaccines. Nat. Med. *14*, 819–821.

Draper, S.J., Biswas, S., Spencer, A.J., Remarque, E.J., Capone, S., Naddeo, M., Dicks, M.D.J., Faber, B.W., de Cassan, S.C., Folgori, A., et al. (2010). Enhancing blood-stage malaria subunit vaccine immunogenicity in rhesus macaques by combining adenovirus, poxvirus, and protein-in-adjuvant vaccines. J. Immunol. *185*, 7583–7595.

Duncan, C.J., Hill, A.V., and Ellis, R.D. (2012). Can growth inhibition assays (GIA) predict blood-stage malaria vaccine efficacy? Hum. Vaccin. Immunother. *8*, 706–714.

Durocher, Y., Perret, S., and Kamen, A. (2002). High-level and high-throughput recombinant protein production by transient transfection of suspension-growing human 293-EBNA1 cells. Nucleic Acids Res. *30*, E9.

Dutta, S., Sullivan, J.S., Grady, K.K., Haynes, J.D., Komisar, J., Batchelor, A.H., Soisson, L., Diggs, C.L., Heppner, D.G., Lanar, D.E., et al. (2009). High antibody titer against apical membrane antigen-1 is required to protect against malaria in the Aotus model. PLoS ONE *4*, e8138.

Elias, S.C., Choudhary, P., de Cassan, S.C., Biswas, S., Collins, K.A., Halstead, F.D., Bliss, C.M., Ewer, K.J., Hodgson, S.H., Duncan, C.J., et al. (2014). Analysis of human B-cell responses following ChAd63-MVA MSP1 and AMA1 immunization and controlled malaria infection. Immunology *141*, 628–644.

Ewer, K.J., O'Hara, G.A., Duncan, C.J., Collins, K.A., Sheehy, S.H., Reyes-Sandoval, A., Goodman, A.L., Edwards, N.J., Elias, S.C., Halstead, F.D., et al. (2013). Protective CD8+ T-cell immunity to human malaria induced by chimpanzee adenovirus-MVA immunisation. Nat. Commun. *4*, 2836.

Foquet, L., Hermsen, C.C., van Gemert, G.J., Van Braeckel, E., Weening, K.E., Sauerwein, R., Meuleman, P., and Leroux-Roels, G. (2014). Vaccine-induced monoclonal antibodies targeting circumsporozoite protein prevent Plasmodium falciparum infection. J. Clin. Invest. *124*, 140–144.

Goodman, A.L., and Draper, S.J. (2010). Blood-stage malaria vaccines - recent progress and future challenges. Ann. Trop. Med. Parasitol. *104*, 189–211.

Goodman, A.L., Epp, C., Moss, D., Holder, A.A., Wilson, J.M., Gao, G.P., Long, C.A., Remarque, E.J., Thomas, A.W., Ammendola, V., et al. (2010). New candidate vaccines against blood-stage Plasmodium falciparum malaria: prime-boost immunization regimens incorporating human and simian adenoviral vectors and poxviral vectors expressing an optimized antigen based on merozoite surface protein 1. Infect. Immun. *78*, 4601–4612.

Goodman, A.L., Forbes, E.K., Williams, A.R., Douglas, A.D., de Cassan, S.C., Bauza, K., Biswas, S., Dicks, M.D., Llewellyn, D., Moore, A.C., et al. (2013). The utility of Plasmodium berghei as a rodent model for anti-merozoite malaria vaccine assessment. Sci Rep *3*, 1706.

Gupta, S., Snow, R.W., Donnelly, C.A., Marsh, K., and Newbold, C. (1999). Immunity to non-cerebral severe malaria is acquired after one or two infections. Nat. Med. *5*, 340–343.

Hayton, K., Gaur, D., Liu, A., Takahashi, J., Henschen, B., Singh, S., Lambert, L., Furuya, T., Bouttenot, R., Doll, M., et al. (2008). Erythrocyte binding protein PfRH5 polymorphisms determine species-specific pathways of Plasmodium falciparum invasion. Cell Host Microbe *4*, 40–51.

Hayton, K., Dumoulin, P., Henschen, B., Liu, A., Papakrivos, J., and Wellems, T.E. (2013). Various PfRH5 polymorphisms can support Plasmodium falciparum invasion into the erythrocytes of owl monkeys and rats. Mol. Biochem. Parasitol. *187*, 103–110.

Hill, A.V. (2011). Vaccines against malaria. Philos. Trans. R. Soc. Lond. B Biol. Sci. *366*, 2806–2814.

Hisaeda, H., Saul, A., Reece, J.J., Kennedy, M.C., Long, C.A., Miller, L.H., and Stowers, A.W. (2002). Merozoite surface protein 3 and protection against malaria in Aotus nancymai monkeys. J. Infect. Dis. *185*, 657–664.

Hodgson, S.H., Choudhary, P., Elias, S.C., Milne, K.H., Rampling, T.W., Biswas, S., Poulton, I.D., Miura, K., Douglas, A.D., Alanine, D.G., et al. (2014). Combining Viral Vectored and Protein-in-adjuvant Vaccines Against the Blood-stage Malaria Antigen AMA1: Report on a Phase 1a Clinical Trial. Mol. Ther. *22*, 2142–2154.

Jones, T.R., Narum, D.L., Gozalo, A.S., Aguiar, J., Fuhrmann, S.R., Liang, H., Haynes, J.D., Moch, J.K., Lucas, C., Luu, T., et al. (2001). Protection of Aotus monkeys by Plasmodium falciparum EBA-175 region II DNA prime-protein boost immunization regimen. J. Infect. Dis. *183*, 303–312.

Kennedy, M.C., Wang, J., Zhang, Y., Miles, A.P., Chitsaz, F., Saul, A., Long, C.A., Miller, L.H., and Stowers, A.W. (2002). In vitro studies with recombinant Plasmodium falciparum apical membrane antigen 1 (AMA1): production and activity of an AMA1 vaccine and generation of a multiallelic response. Infect. Immun. *70*, 6948–6960.

Kester, K.E., Cummings, J.F., Ofori-Anyinam, O., Ockenhouse, C.F., Krzych, U., Moris, P., Schwenk, R., Nielsen, R.A., Debebe, Z., Pinelis, E., et al.; RTS,S Vaccine Evaluation Group (2009). Randomized, double-blind, phase 2a trial of falciparum malaria vaccines RTS,S/AS01B and RTS,S/AS02A in malaria-naive adults: safety, efficacy, and immunologic associates of protection. J. Infect. Dis. *200*, 337–346.

Lyon, J.A., Angov, E., Fay, M.P., Sullivan, J.S., Girourd, A.S., Robinson, S.J., Bergmann-Leitner, E.S., Duncan, E.H., Darko, C.A., Collins, W.E., et al. (2008). Protection induced by Plasmodium falciparum MSP1(42) is strain-specific, antigen and adjuvant dependent, and correlates with antibody responses. PLoS ONE *3*, e2830.

Mahdi Abdel Hamid, M., Remarque, E.J., van Duivenvoorde, L.M., van der Werff, N., Walraven, V., Faber, B.W., Kocken, C.H., and Thomas, A.W. (2011). Vaccination with Plasmodium knowlesi AMA1 formulated in the novel adjuvant co-vaccine HT™ protects against blood-stage challenge in rhesus macaques. PLoS ONE *6*, e20547.

Manske, M., Miotto, O., Campino, S., Auburn, S., Almagro-Garcia, J., Maslen, G., O'Brien, J., Djimde, A., Doumbo, O., Zongo, I., et al. (2012). Analysis of Plasmodium falciparum diversity in natural infections by deep sequencing. Nature *487*, 375–379.

Miura, K., Zhou, H., Diouf, A., Moretz, S.E., Fay, M.P., Miller, L.H., Martin, L.B., Pierce, M.A., Ellis, R.D., Mullen, G.E., and Long, C.A. (2009). Anti-apical-membrane-antigen-1 antibody is more effective than anti-42-kilodalton-merozoite-surface-protein-1 antibody in inhibiting plasmodium falciparum growth, as determined by the in vitro growth inhibition assay. Clin. Vaccine Immunol. *16*, 963–968.

Moorthy, V.S., Newman, R.D., and Okwo-Bele, J.M. (2013). Malaria vaccine technology roadmap. Lancet *382*, 1700–1701.

Murray, C.J., Rosenfeld, L.C., Lim, S.S., Andrews, K.G., Foreman, K.J., Haring, D., Fullman, N., Naghavi, M., Lozano, R., and Lopez, A.D. (2012). Global malaria mortality between 1980 and 2010: a systematic analysis. Lancet *379*, 413–431.

Murungi, L.M., Kamuyu, G., Lowe, B., Bejon, P., Theisen, M., Kinyanjui, S.M., Marsh, K., and Osier, F.H. (2013). A threshold concentration of anti-merozoite antibodies is required for protection from clinical episodes of malaria. Vaccine *31*, 3936–3942.

Patel, S.D., Ahouidi, A.D., Bei, A.K., Dieye, T.N., Mboup, S., Harrison, S.C., and Duraisingh, M.T. (2013). Plasmodium falciparum merozoite surface antigen, PfRH5, elicits detectable levels of invasion-inhibiting antibodies in humans. J. Infect. Dis. *208*, 1679–1687.

Plotkin, S.A. (2010). Correlates of protection induced by vaccination. Clin. Vaccine Immunol. *17*, 1055–1065.

Reddy, K.S., Pandey, A.K., Singh, H., Sahar, T., Emmanuel, A., Chitnis, C.E., Chauhan, V.S., and Gaur, D. (2014). Bacterially expressed full-length recombinant Plasmodium falciparum RH5 protein binds erythrocytes and elicits potent strain-transcending parasite-neutralizing antibodies. Infect. Immun. *82*, 152–164.

Remarque, E.J., Faber, B.W., Kocken, C.H., and Thomas, A.W. (2008). Apical membrane antigen 1: a malaria vaccine candidate in review. Trends Parasitol. *24*, 74–84.

Riley, E.M., and Stewart, V.A. (2013). Immune mechanisms in malaria: new insights in vaccine development. Nat. Med. *19*, 168–178.

Sheehy, S.H., Duncan, C.J., Elias, S.C., Collins, K.A., Ewer, K.J., Spencer, A.J., Williams, A.R., Halstead, F.D., Moretz, S.E., Miura, K., et al. (2011). Phase Ia clinical evaluation of the Plasmodium falciparum blood-stage antigen MSP1 in ChAd63 and MVA vaccine vectors. Mol. Ther. *19*, 2269–2276.

Sheehy, S.H., Duncan, C.J., Elias, S.C., Choudhary, P., Biswas, S., Halstead, F.D., Collins, K.A., Edwards, N.J., Douglas, A.D., Anagnostou, N.A., et al. (2012). ChAd63-MVA-vectored blood-stage malaria vaccines targeting MSP1 and AMA1: assessment of efficacy against mosquito bite challenge in humans. Mol. Ther. *20*, 2355–2368.

Sheehy, S.H., Douglas, A.D., and Draper, S.J. (2013). Challenges of assessing the clinical efficacy of asexual blood-stage Plasmodium falciparum malaria vaccines. Hum. Vaccin. Immunother. *9*, 1831–1840.

Singh, S., Miura, K., Zhou, H., Muratova, O., Keegan, B., Miles, A., Martin, L.B., Saul, A.J., Miller, L.H., and Long, C.A. (2006). Immunity to recombinant plasmodium falciparum merozoite surface protein 1 (MSP1): protection in Aotus nancymai monkeys strongly correlates with anti-MSP1 antibody titer and in vitro parasite-inhibitory activity. Infect. Immun. *74*, 4573–4580.

Spring, M.D., Cummings, J.F., Ockenhouse, C.F., Dutta, S., Reidler, R., Angov, E., Bergmann-Leitner, E., Stewart, V.A., Bittner, S., Juompan, L., et al. (2009). Phase 1/2a study of the malaria vaccine candidate apical membrane antigen-1 (AMA-1) administered in adjuvant system AS01B or AS02A. PLoS ONE *4*, e5254.

Stowers, A.W., and Miller, L.H. (2001). Are trials in New World monkeys on the critical path for blood-stage malaria vaccine development? Trends Parasitol. *17*, 415–419.

Stowers, A.W., Cioce, V., Shimp, R.L., Lawson, M., Hui, G., Muratova, O., Kaslow, D.C., Robinson, R., Long, C.A., and Miller, L.H. (2001). Efficacy of two alternate vaccines based on Plasmodium falciparum merozoite surface protein 1 in an Aotus challenge trial. Infect. Immun. *69*, 1536–1546.

Stowers, A.W., Kennedy, M.C., Keegan, B.P., Saul, A., Long, C.A., and Miller, L.H. (2002). Vaccination of monkeys with recombinant Plasmodium falciparum apical membrane antigen 1 confers protection against blood-stage malaria. Infect. Immun. *70*, 6961–6967.

Thera, M.A., Doumbo, O.K., Coulibaly, D., Laurens, M.B., Ouattara, A., Kone, A.K., Guindo, A.B., Traore, K., Traore, I., Kouriba, B., et al. (2011). A field trial to assess a blood-stage malaria vaccine. N. Engl. J. Med. *365*, 1004–1013.

Tran, T.M., Ongoiba, A., Coursen, J., Crosnier, C., Diouf, A., Huang, C.Y., Li, S., Doumbo, S., Doumtabe, D., Kone, Y., et al. (2014). Naturally acquired antibodies specific for Plasmodium falciparum reticulocyte-binding protein homologue 5 inhibit parasite growth and predict protection from malaria. J. Infect. Dis. *209*, 789–798.

Villasis, E., Lopez-Perez, M., Torres, K., Gamboa, D., Neyra, V., Bendezu, J., Tricoche, N., Lobo, C., Vinetz, J.M., and Lustigman, S. (2012). Anti-Plasmodium falciparum invasion ligand antibodies in a low malaria transmission region, Loreto, Peru. Malar. J. *11*, 361.

Wanaguru, M., Liu, W., Hahn, B.H., Rayner, J.C., and Wright, G.J. (2013). RH5-Basigin interaction plays a major role in the host tropism of Plasmodium falciparum. Proc. Natl. Acad. Sci. USA *110*, 20735–20740.

Weiss, W.R., Kumar, A., Jiang, G., Williams, J., Bostick, A., Conteh, S., Fryauff, D., Aguiar, J., Singh, M., O'Hagan, D.T., et al. (2007). Protection of rhesus monkeys by a DNA prime/poxvirus boost malaria vaccine depends on optimal DNA priming and inclusion of blood stage antigens. PLoS ONE *2*, e1063.

Williams, A.R., Douglas, A.D., Miura, K., Illingworth, J.J., Choudhary, P., Murungi, L.M., Furze, J.M., Diouf, A., Miotto, O., Crosnier, C., et al. (2012). Enhancing blockade of Plasmodium falciparum erythrocyte invasion: assessing combinations of antibodies against PfRH5 and other merozoite antigens. PLoS Pathog. *8*, e1002991.

Wright, G.J., and Rayner, J.C. (2014). Plasmodium falciparum erythrocyte invasion: combining function with immune evasion. PLoS Pathog. *10*, e1003943.

Cell Host & Microbe, Volume *17*

Supplemental Information

A PfRH5-Based Vaccine Is Efficacious against Heterologous Strain Blood-Stage *Plasmodium falciparum* Infection in *Aotus* Monkeys

Alexander D. Douglas, G. Christian Baldeviano, Carmen M. Lucas, Luis A. Lugo-Roman, Cécile Crosnier, S. Josefin Bartholdson, Ababacar Diouf, Kazutoyo Miura, Lynn E. Lambert, Julio A. Ventocilla, Karina P. Leiva, Kathryn H. Milne, Joseph J. Illingworth, Alexandra J. Spencer, Kathryn A. Hjerrild, Daniel G.W. Alanine, Alison V. Turner, Jeromy T. Moorhead, Kimberly A. Edgel, Yimin Wu, Carole A. Long, Gavin J. Wright, Andrés G. Lescano, and Simon J. Draper

Figure S1

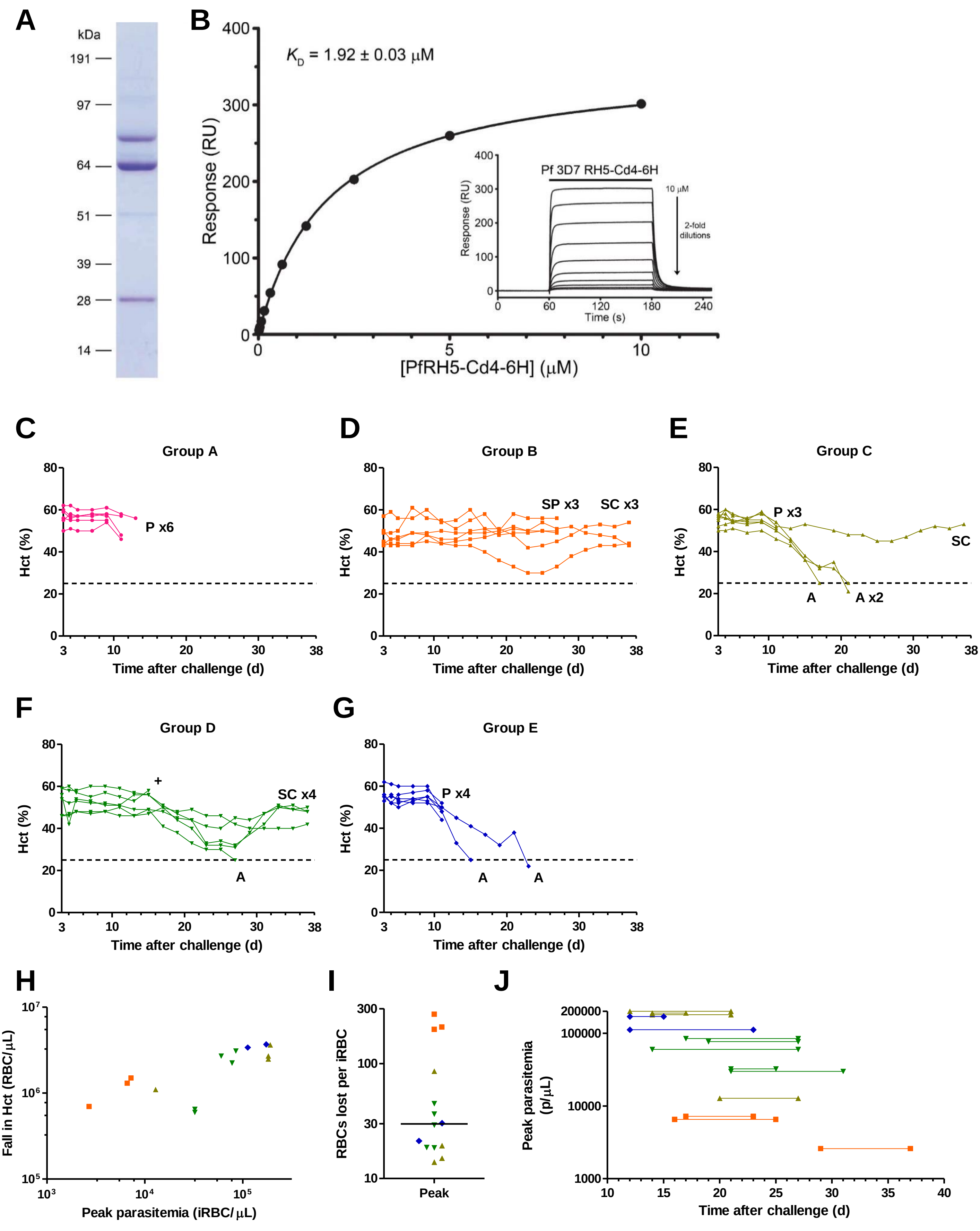

A
kDa
191
97
64
51
39
28
14
B
K_D = 1.92 ± 0.03 μM
Response (RU)
[PfRH5-Cd4-6H] (μM)
Pf 3D7 RH5-Cd4-6H
10 μM
2-fold dilutions
Time (s)
C
Group A
P x6
D
Group B
SP x3
SC x3
E
Group C
P x3
SC
A
A x2
F
Group D
+
SC x4
A
G
Group E
P x4
A
A
Hct (%)
Time after challenge (d)
H
Fall in Hct (RBC/μL)
Peak parasitemia (iRBC/μL)
I
RBCs lost per iRBC
Peak
J
Peak parasitemia (p/μL)
Time after challenge (d)

Figure S2

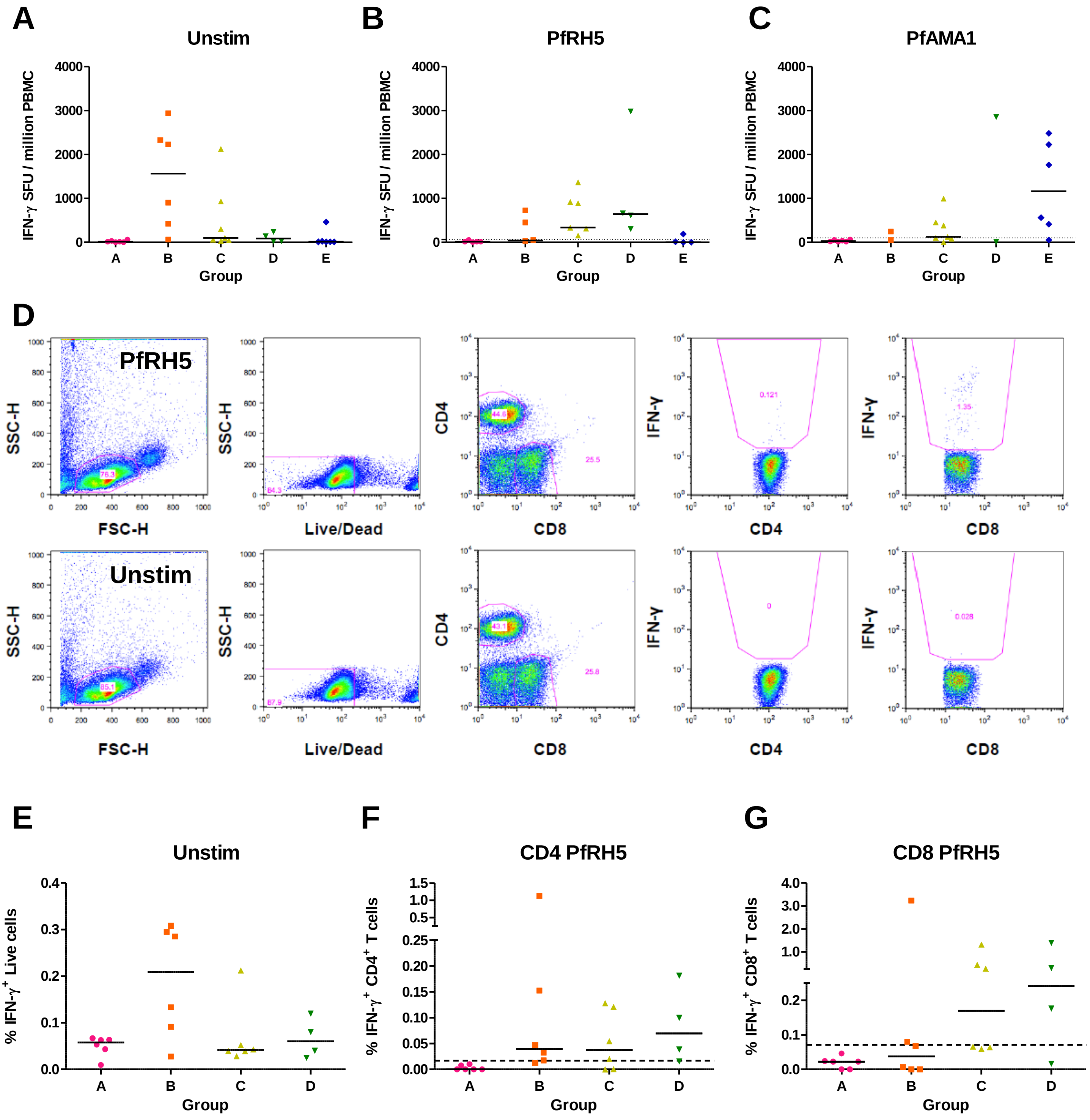
A
Unstim
IFN-γ SFU / million PBMC
Group
B
PfRH5
C
PfAMA1
D
PfRH5
Unstim
SSC-H
FSC-H
Live/Dead
CD4
CD8
IFN-γ
E
Unstim
% IFN-γ+ Live cells
F
CD4 PfRH5
% IFN-γ+ CD4+ T cells
G
CD8 PfRH5
% IFN-γ+ CD8+ T cells

Figure S3

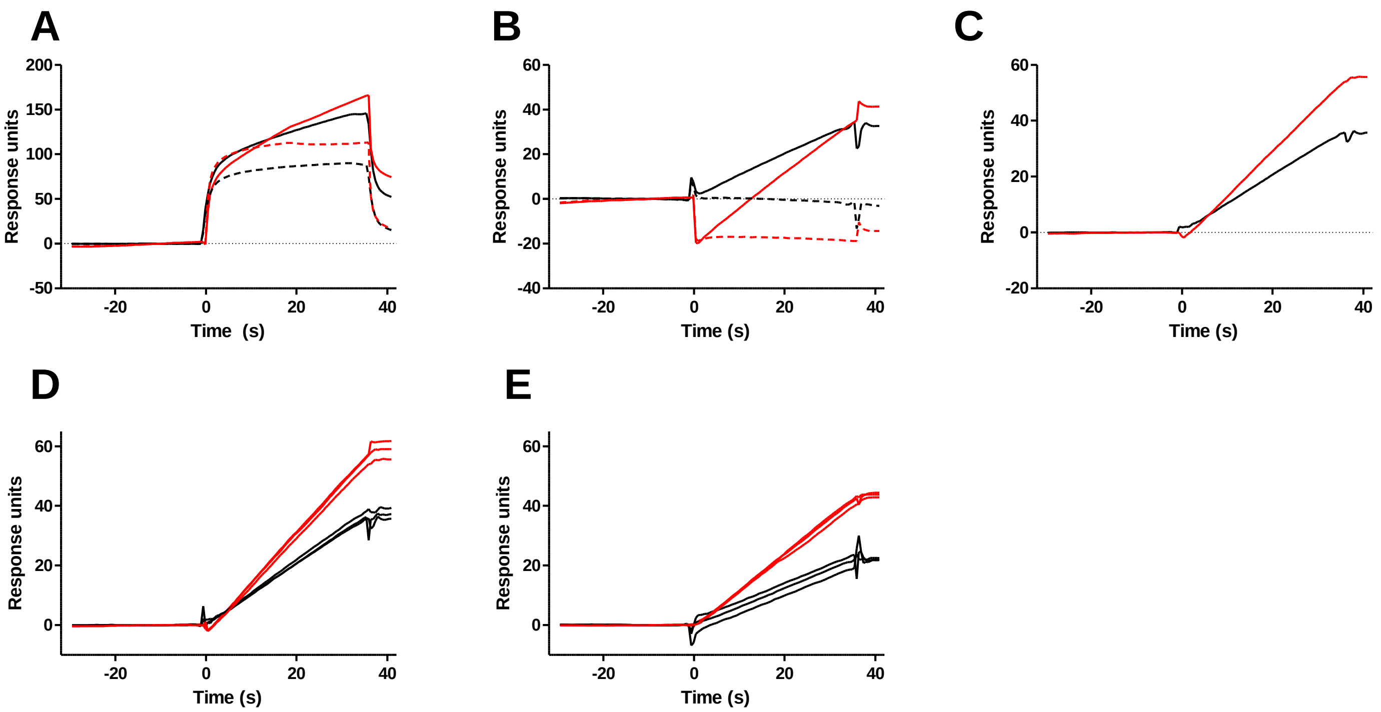

F

	Replicate	Calculated Concentration (µg/mL)	Fitted initial binding rate (RU/s)		QC ratio fit	Chi² (RU²)	SE (calc. conc)
			5µL/min flow	100µL/min flow			
PfRH5 (Group D pool)	1	330	*1.04*	*1.67*	*0.352*	*0.17*	1.5
	2	380	*1.12*	*1.73*	*0.319*	*0.0077*	0.75
	3	330	*1.02*	*1.64*	*0.347*	*0.0048*	0.5125
	**Mean**	**347**					
PfAMA1 (Group E pool)	1	150	*0.62*	*1.23*	*0.579*	*0.01*	0.325
	2	160	*0.628*	*1.17*	*0.503*	*0.037*	0.7375
	3	150	*0.623*	*1.24*	*0.58*	*0.062*	0.425
	**Mean**	**153**					

Figure S4

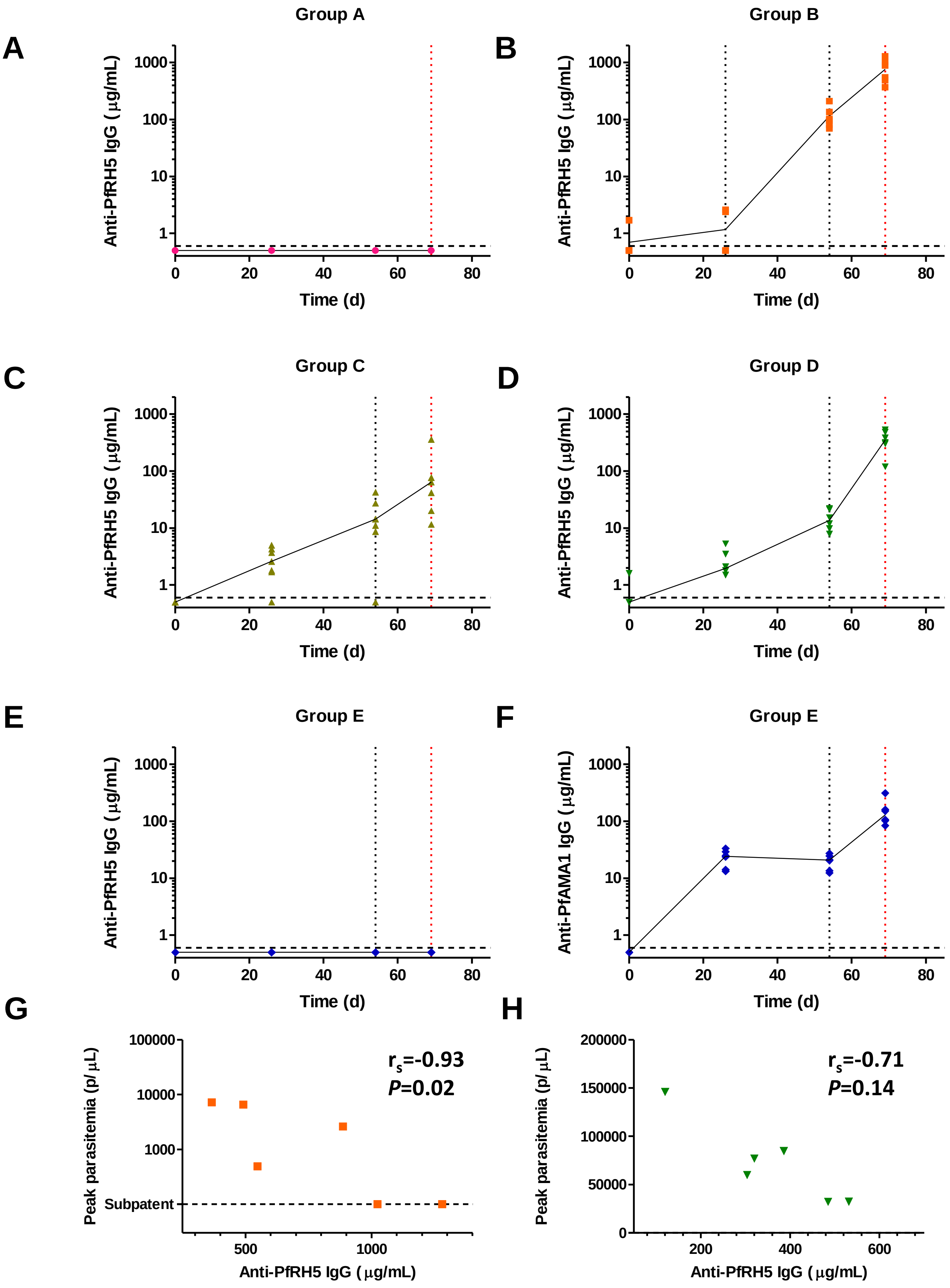

A
Group A
Anti-PfRH5 IgG (µg/mL)
1000
100
10
1
0
20
40
60
80
Time (d)
B
Group B
C
Group C
D
Group D
E
Group E
F
Group E
Anti-PfAMA1 IgG (µg/mL)
G
Peak parasitemia (p/µL)
100000
10000
1000
Subpatent
500
1000
Anti-PfRH5 IgG (µg/mL)
r_s=-0.93
P=0.02
H
200000
150000
100000
50000
0
200
400
600
r_s=-0.71
P=0.14

Figure S5

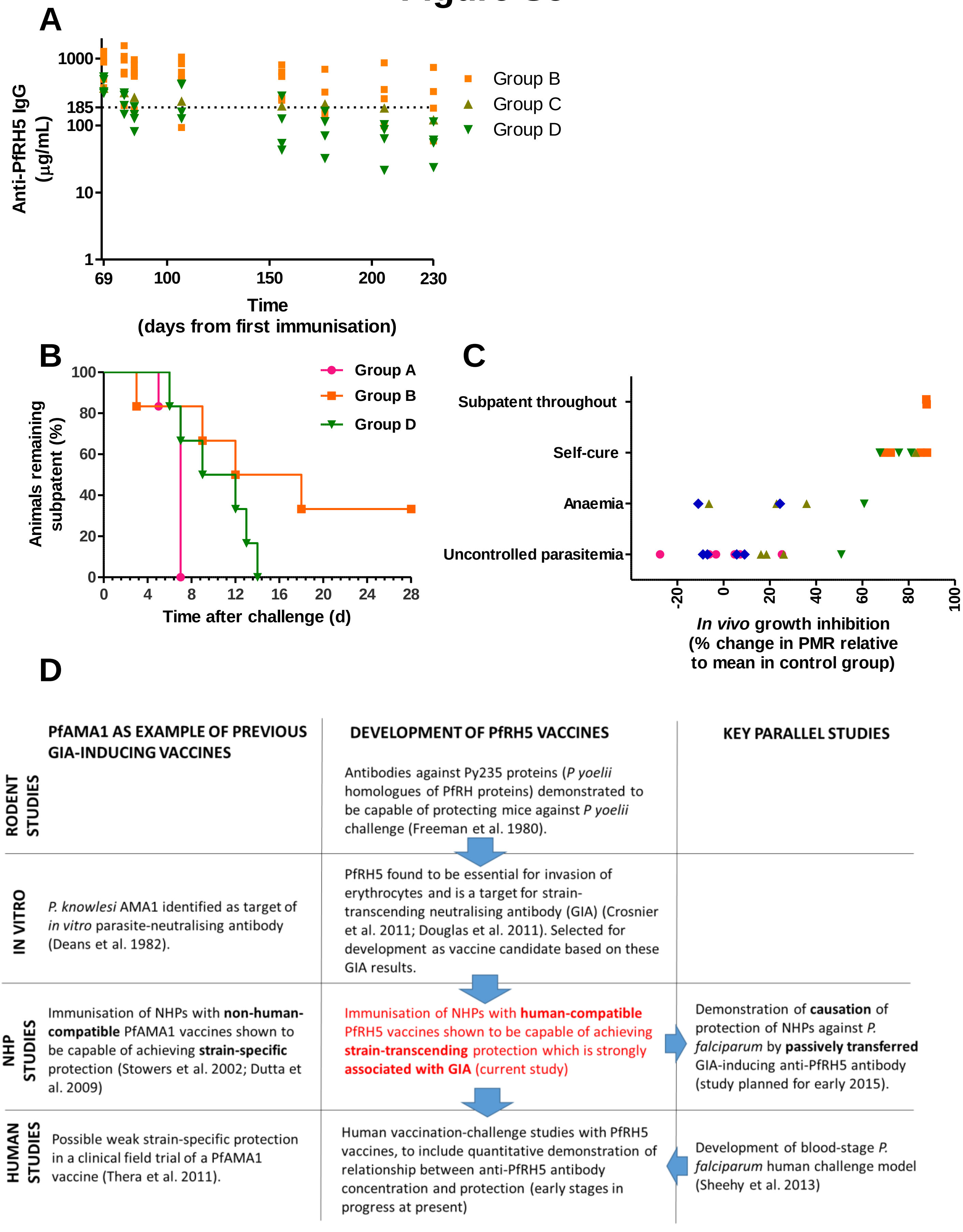
A
Anti-PfRH5 IgG (µg/mL)
1000
185
100
10
1
69
100
150
200
230
Time (days from first immunisation)
Group B
Group C
Group D
B
Animals remaining subpatent (%)
100
80
60
40
20
0
4
8
12
16
20
24
28
Time after challenge (d)
Group A
Group B
Group D
C
Subpatent throughout
Self-cure
Anaemia
Uncontrolled parasitemia
-20
0
20
40
60
80
100
In vivo growth inhibition (% change in PMR relative to mean in control group)
D
PfAMA1 AS EXAMPLE OF PREVIOUS GIA-INDUCING VACCINES
DEVELOPMENT OF PfRH5 VACCINES
KEY PARALLEL STUDIES
RODENT STUDIES
Antibodies against Py235 proteins (P yoelii homologues of PfRH proteins) demonstrated to be capable of protecting mice against P yoelii challenge (Freeman et al. 1980).
IN VITRO
P. knowlesi AMA1 identified as target of in vitro parasite-neutralising antibody (Deans et al. 1982).
PfRH5 found to be essential for invasion of erythrocytes and is a target for strain-transcending neutralising antibody (GIA) (Crosnier et al. 2011; Douglas et al. 2011). Selected for development as vaccine candidate based on these GIA results.
NHP STUDIES
Immunisation of NHPs with non-human-compatible PfAMA1 vaccines shown to be capable of achieving strain-specific protection (Stowers et al. 2002; Dutta et al. 2009)
Immunisation of NHPs with human-compatible PfRH5 vaccines shown to be capable of achieving strain-transcending protection which is strongly associated with GIA (current study)
Demonstration of causation of protection of NHPs against P. falciparum by passively transferred GIA-inducing anti-PfRH5 antibody (study planned for early 2015).
HUMAN STUDIES
Possible weak strain-specific protection in a clinical field trial of a PfAMA1 vaccine (Thera et al. 2011).
Human vaccination-challenge studies with PfRH5 vaccines, to include quantitative demonstration of relationship between anti-PfRH5 antibody concentration and protection (early stages in progress at present)
Development of blood-stage P. falciparum human challenge model (Sheehy et al. 2013)

SUPPLEMENTAL FIGURE LEGENDS

Figure S1, related to Figure 1: Production of PfRH5 protein vaccine and analysis of haematocrit in challenged animals

(A) Coomassie-stained SDS-PAGE gel of the PfRH5 protein vaccine immunogen run under reducing conditions. The protein is the 3D7 allele of PfRH5 with a C-terminal fusion of rat Cd4 domains 3 and 4 followed by a hexa-histidine tag (PfRH5-Cd4-His6). As previously described (Bustamante et al., 2013), two principle bands are present, at approximately 85kDa and 65kDa. The 85kDa band represents the full-length PfRH5 protein (66kDa) plus the C-terminal tags, with the 65kDa species constituting a 45kDa processing product of PfRH5 plus tags, following loss of the N-terminus (similar to the processing product observed in parasite culture supernatant (Baum et al., 2009)). The 28kDa species is primarily cleaved Cd4 tag (data not shown). **(B)** Equilibrium binding analysis of PfRH5-Cd4-His6 to its receptor basigin, quantified using surface plasmon resonance (SPR). Serial dilutions of the purified PfRH5-Cd4-His6 protein preparation (used for *Aotus* immunizations) were injected over the basigin protein immobilized on a sensor chip. Reference-subtracted curves are shown, demonstrating that equilibrium was reached (inset) and that binding was saturable. An equilibrium dissociation constant of 1.92 ± 0.03 µM was calculated from the binding data (see Methods).

(C-G) Timecourse of hematocrit (Hct) for individual animals in Groups A-E respectively. Horizontal dotted lines indicate 25% Hct threshold for initiation of antimalarial treatment because of anaemia. Symbols as per Figure 1B-E. **(H)** Relationship between peak parasitemia and RBC/µL 'lost' due to malaria among the 14 animals which self-controlled microscopically-patent parasitemia (i.e. excluding animals treated for hyperparasitemia and the 3 animals in Group B with ≤1 microscopically observed parasite throughout the study; calculation based upon 10^7 cells per µL packed RBCs). A significant correlation was observed (Spearman's r_S=0.76, *P*=0.002). **(I)** A median of 30 'lost' RBC per iRBC was observed at the peak of parasitemia [data shown in panel H, represented as fall in Hct divided by peak parasitemia]. **(J)** For each of the 14 parasite-controlling animals, a line at the level of peak parasitemia

(y-axis) extends from the time of peak parasitemia (left end of line) to the time of minimum Hct (right end of line), illustrating the median delay of 8 days between these events. Among these 14 animals, significant negative correlations were observed between magnitude of peak parasitemia and i) timing of peak parasitemia (Spearman's r_s=-0.73, *P*=0.003) and ii) timing of minimum Hct (Spearman's r_s=-0.69, *P*=0.007), such that animals controlling parasitemia at a lower level did so later, and had later nadirs in their Hct. These results are suggestive of destruction of uninfected erythrocytes, which may be immunologically mediated.

Figure S2, related to Figure 2: ELISpot and ICS responses on day of challenge.

Ex-vivo IFN-γ ELISpot was conducted from frozen PBMC samples collected on day 69 (day of challenge; DoC). Cells from all animals produced >1000 spot forming units (SFU) per million PBMC in response to positive control SEB/PHA stimulation (not shown). Insufficient cells were available for two animals from Group D. **(A)** Individual responses (mean of duplicate wells) and group median responses are plotted from the unstimulated control wells. High levels of non-peptide-specific responses were consistently seen in animals from Group B, possibly due to Freund's adjuvant-induced innate cell activation. Individual responses (mean of duplicate wells) and group median responses are plotted for **(B)** PfRH5 peptide pool-stimulated and **(C)** PfAMA1 peptide pool-stimulated wells after subtraction of responses in negative control unstimulated wells; horizontal dashed line indicates mean plus three standard deviations (SD) of the apparent response in Group A animals (peptide stimulated minus unstimulated). Mainly PfRH5-specific responses were seen in animals receiving a viral vectored PfRH5 vaccine in Groups C and D, whilst PfAMA1-specific responses were similarly observed in Group E. Unexpectedly some animals in Groups C and D appeared to have PfAMA1-specific responses; the reason for this is unclear, but it is possible this may reflect previous exposure of these animals to environmental apicomplexa. **(D-G)** ICS was conducted on frozen PBMC samples. Cells were as for the ELISpot assay with the exception that insufficient cells were available for one further animal (from Group C). **(D)** Gating strategy for a representative sample showing the PfRH5 peptide pool-stimulated response (top row) and unstimulated control (bottom row). Lymphocytes were gated, followed by live cells, then $CD4^+$ or $CD8^+$ subsets, followed by IFN-γ^+ cells within each subset. **(E)** The % of live IFN-γ^+ cells in the lymphocyte gate was assessed. Individual responses and group median are plotted. As with ELISpot, the highest levels of non-specific IFN-γ were detected in Group B. PfRH5-specific responses as the % of **(F)** live $CD4^+$ cells and **(G)** $CD8^+$ cells which are IFN-γ^+ in PfRH5 peptide pool-stimulated wells, after subtraction of responses in negative control unstimulated wells. Individual responses (points) and group median responses (lines) are plotted; horizontal dashed line as for panels B-C.

Figure S3, related to Figure 3: Calibration free concentration analysis (CFCA) of antibody responses.

CFCA was performed using a Biacore T200 instrument in order to measure absolute (µg/mL) concentrations of antigen-specific antibody in pooled plasma collected on day 69 (DoC) from Group D (anti-PfRH5) and Group E (anti-PfAMA1). Throughout, binding at low sample flow rate (5µL/min) is indicated by black lines while binding at high flow (100µL/min) is indicated by red lines.

(A-C) depict processing of the data used to make a single measurement of anti-PfRH5 IgG concentration. **(A)** Binding of PfRH5-immune plasma (Group D pool, solid lines) and control plasma (Group A pool, dashed lines) to the PfRH5-coated flow cell (Fc2) at the two flow rates, as above. **(B)** Binding of PfRH5-immune and control plasma at the two flow rates, after subtraction of binding to the non-PfRH5 coated reference flow cell (i.e. graph shows Fc2-1). **(C)** Final double-subtracted PfRH5-specific binding at the two flow rates, after subtraction of control sample binding from reference-subtracted PfRH5-immune sample binding (i.e. graph shows difference between solid and dashed lines in **(B)**). The slopes of these lines, as estimated by the CFCA function in the T200 evaluation software, were used to calculate antigen-specific antibody concentration in the PfRH5-immune sample.

(D) and **(E)** depict double-subtracted results of replicate measurements of **(D)** PfRH5-specific binding in the Group D pool and **(E)** PfAMA1-specific binding in the Group E pool.

(F) Tabulated overall CFCA results, indicating calculated concentration of 347 µg/mL PfRH5-specific antibody in the Group D pool and 153 µg/mL PfAMA1-specific antibody in the Group E pool. SE = standard error of fit.

Figure S4, related to Figure 3: ELISA timecourses and within-group association of protection with pre-challenge anti-PfRH5 antibody concentration.

(A-F) Anti-PfRH5 and anti-PfAMA1 plasma total IgG responses were monitored over time by ELISA. Plots show the results for each individual animal, and the joining black line links the medians at each time-point. All animals received their first vaccine on day 0. The black vertical dashed lines indicate booster immunization time-points for each group, and the red vertical dashed line indicates the DoC. Plasma were tested at days 0, 28, 54 and 69 (DoC). **(A-E)** Anti-PfRH5 (3D7) responses for Groups A-E respectively, and **(F)** Anti-PfAMA1 (FVO) responses for Group E. Arbitrary ELISA units were converted to µg/mL titers following definition of a conversion factor by CFCA.

Panels **(G-H)** show relationship between DoC plasma antibody concentration and challenge outcome within **(G)** Group B, and **(H)** Group D. For each group, Spearman's rank correlation coefficient (r_s) and *P* value are shown for the relationship of anti-PfRH5 total IgG concentration on DoC with peak parasitemia.

Figure S5, related to Figures 1, 3, 4 and 5: Impact of PfRH5 vaccines upon Phase IIa CHMI end-points, long-term maintenance of anti-PfRH5 antibody concentrations after challenge, and context of current study relative to other experiments.

(A) ELISA measured antibody concentrations between day of challenge and day 230 of the study (day 161 after challenge) are shown for animals which self-cured infection. Dotted horizontal line indicates estimated IVGI EC_{50} of 185 µg/mL.

(B) Kaplan-Meier plot of % animals remaining with microscopically sub-patent parasitemia (subjects in Phase IIa controlled human malaria infection (CHMI) trials must be treated at the time of microscopic patency (Sauerwein et al., 2011)). For clarity, this includes only the control group (A) and the two groups (B and D) for which vaccine efficacy was significant as assessed by the primary efficacy endpoint (Figure 1G). Comparing sub-patent survival to Group A by Mann-Whitney test without correction for multiple comparisons, *P*=0.06 for Group B and *P*=0.09 for Group D. As an alternative to measuring time to microscopically patent parasitemia in CHMI studies, PMR can be measured by curve-fitting to PCR-quantified sub-patent parasitaemia (Douglas et al., 2013). **(C)** Challenge outcome versus IVGI, i.e. the percentage reduction in the PMR in each animal relative to the mean in the control group. PMR reductions in excess of 65% appear to be required to enable these malaria-naïve *Aotus* to survive blood-stage *P. falciparum* FVO strain challenge without treatment. Such large changes in PMR would be readily detectable after CHMI (Sanderson et al., 2008), supporting the assertion that efficacious blood-stage vaccines should be able to demonstrate *in vivo* biological effects in CHMI trials prior to field trials (Sheehy et al., 2013).

(D) Context of the data reported in this manuscript as related to past and future studies; the future studies referred to will be necessary for validation of vaccine-induced GIA as an achievable mechanism of protection of humans against *P. falciparum.* Manuscripts cited are as follows. (Crosnier et al., 2011; Deans et al., 1982; Douglas et al., 2011; Dutta et al., 2009; Freeman et al., 1980; Sheehy et al., 2013; Stowers et al., 2002; Thera et al., 2011).

SUPPLEMENTAL TABLE

Table S1, related to Figures 1 and S1: A review of previous *Aotus nancymaae* – *P. falciparum* challenge studies.

Fifty-five *Aotus nancymaae* monkeys have previously been challenged with 10^4 FVO parasites after control immunizations using Freund's adjuvant; not a single one of these has survived the challenge without treatment.

SUPPLEMENTAL METHODS

Vaccines

All PfRH5 vaccines were based upon the *P. falciparum* 3D7 clone sequence (NCBI XM_001351508.1). The production of recombinant adenovirus and modified vaccinia virus Ankara (MVA) viral vector vaccines, expressing full-length PfRH5, has previously been described (Douglas et al., 2011). In this case the PfRH5 transgene encodes amino acids (αα) 26-526, ENAI…PLTQ, with substitutions N38Q and N214Q to remove two sites of potential N-linked glycosylation. For the current study, a chimpanzee adenovirus serotype 63 (ChAd63) expressing the same PfRH5 transgene was produced, using previously described methods (Douglas et al., 2011; Goodman et al., 2010; Goodman et al., 2013). The production of the ChAd63 PfAMA1 vaccine (expressing a bi-allelic transgene encoding the 3D7 and FVO strain sequences) and of the PfAMA1 protein based upon the FVO allele have been reported elsewhere (Biswas et al., 2011; Kennedy et al., 2002). Recombinant ChAd63 expressing *Renilla* luciferase (Orubu et al., 2012) was produced using the same methods and a transgene insert as previously described (Dicks MDJ *et al*., manuscript in preparation).

The PfRH5 3D7 protein was expressed essentially as described (Crosnier et al., 2011). Briefly, the full-length 3D7 PfRH5 coding sequence was expressed as a secreted recombinant protein following transient transfection of HEK293E cells (Bushell et al., 2008; Durocher et al., 2002). The PfRH5 coding sequence was synthesized (Geneart GmbH, Regensburg, Germany) and codon optimized for expression in human cells. The protein was fused in-frame at the C-terminus to rat CD4 domains 3 and 4 (CD4d3+4) followed by a hexa-histidine (His6) tag for detection and purification. To prevent inappropriate glycosylation when expressed in mammalian cells, the four threonine residues in the context of potential N-linked glycosylation sites (N-X-S/T) were mutated to alanine (T40A, T216A, T286A and T299A) and the signal peptide was replaced with a high-scoring exogenous signal peptide from a mouse light chain antibody (Crosnier et al., 2010). The supernatant was harvested five days post-transfection and cellular debris removed by 0.2 μm filtration. Imidazole was added to a final

concentration of 10mM and NaCl supplemented to a final concentration of 500mM, and the protein was purified using a HiTrap Ni^{2+}-NTA Sepharose column (GE Healthcare) and eluted with 400mM imidazole. Eluted fractions containing the purified protein were pooled and the protein was assessed as >90% pure by SDS-PAGE and dialysed three times against 1L of PBS at 4°C for 24 h. Four independent purifications, which were essentially identical to each other as assessed by SDS-PAGE, were combined to create a single 3mL preparation of purified PfRH5-CD4-His6 at a final concentration of 0.9mg/mL (Figure S1A). Three aliquots (one x 1mL and two x 500µL) of the protein were snap-frozen on dry ice and kept frozen during storage and shipping until thawing just prior to immunization. The remaining protein was stored at 4°C and reserved for an additional quality control check for biochemical activity. For this, we used surface plasmon resonance (SPR) as implemented in a T100 BIAcore instrument essentially as described (Crosnier et al., 2011). Briefly, mono-biotinylated basigin-CD4d3+4-biotin was immobilized in a flow cell of a streptavidin-coated sensor chip, with an approximate molar equivalent of a CD4d3+4-biotin control protein used for reference subtraction. Binding data were plotted using the BIAevaluation software supplied by the manufacturer and an equilibrium dissociation constant was calculated using non-linear regression fitting of a simple (1:1) Langmuir binding isotherm to the data (Figure S1B).

Animals, Immunizations, Challenge and Sample Collection

Adult female owl monkeys (*Aotus nancymaae*) were obtained from the Instituto Veterinario de Investigaciones Tropicales y de Altura de la Universidad Nacional Mayor de San Marcos (IVITA-San Marcos University), Peru and transported to the animal facility at US Naval Medical Research Unit No. 6 (NAMRU-6). All animals were quarantined for 4 weeks before the initiation of the study. Randomization to groups was stratified by pre-trial weight. Immunizations were performed by the intramuscular route (into the caudal quadriceps) on days 0 and 54, with the exception of those containing Freund's adjuvant, which were given subcutaneously (into the interscapular area) on days 0 (complete), 26 (incomplete) and 54 (incomplete). Doses used were 5 x 10^9 infectious units (ifu) for

adenoviruses, 2 x 10^8 plaque-forming units (pfu) for MVA, 50µg for protein, 250µL for complete and incomplete Freund's adjuvant (Sigma), and 48µg for Abisco-100 (Isconova), with the final injection volume made up to 500µL with sterile PBS. All immunizations were administered under ketamine anaesthesia.

Groups were as follows (*n*=6/group unless otherwise stated): Group A: sham vaccines – ChAd63 expressing *Renilla* luciferase (RLuc) prime, PBS with Abisco-100 adjuvant boost; Group B: PfRH5 protein with complete or incomplete Freund's adjuvant (CFA, IFA); Group C: ChAd63 expressing PfRH5 prime, PfRH5 protein with Abisco-100 boost (*n*=7); Group D: ChAd63-PfRH5 prime, MVA expressing PfRH5 boost; Group E: ChAd63-PfAMA1 prime, PfAMA1 protein with Abisco-100 boost. Group sizes were chosen using a sample size calculation based upon review of results of previous studies in this model, and reviewed by the University of Oxford Centre for Statistics in Medicine; full details are available on request from the corresponding author.

The study protocol was approved by NAMRU-6's Institutional Animal Care and Use Committee (protocol number NAMRU-6 11-12); the Department of the Navy Bureau of Medicine and Surgery (NRD-748); the University of Oxford Animal Care and Ethical Review Committee; and the Institut Nacional de Recursos Naturales (INRENA) at the Peruvian Ministry of Agriculture. NAMRU-6 is a facility accredited by the Association for Assessment and Accreditation of Laboratory Animal Care (AAALAC); therefore all husbandry and experimental procedures reported herein were conducted in compliance with the Animal Welfare Act and in accordance with the principles set forth in the "Guide for the Care and Use of Laboratory Animals," Institute of Laboratory Animals Resources, National Research Council, National Academy Press, 1996.

15 days after the final vaccination (i.e. study day 69), animals were challenged intravenously with 10^4 FVO-strain *P. falciparum* infected red blood cells (RBC) taken from a donor monkey, as described

(Stowers et al., 2001). From day 72, daily thin-film parasitemia quantification and alternate-day hematocrit (Hct) measurements were conducted. Laboratory staff were blinded to animals' vaccine allocation. Animals were treated when i) parasite density reached ≥200,000/µL; or ii) Hct fell to ≤25%; or iii) upon reaching challenge day 28 (C+28) if no parasites had been seen in the preceding week; or iv) upon reaching C+38 (study day 107). Parasite density (p/µL) was calculated using the following formula, in order to take account of animals' varying hematocrits as well as the percentage parasitemia:

$$\text{Parasite density } = \left(\frac{\text{Percentage parasitemia}}{100}\right) * \left(\frac{\text{Percentage hematocrit}}{100}\right) * 10^7$$

Pre-trial red cell counts (data not shown) were consistent with the value of 10^7 erythrocytes per µL packed cells used in this formula.

Blood samples for immunological assays were collected from all animals' saphenous veins under ketamine anaesthesia on days 0, 26, 54, 69 (day of challenge, DoC), and 107 (C+38). EDTA-anticoagulated blood was prepared using standard methods to obtain plasma and PBMCs.

ELISpot

Ex-vivo interferon-γ (IFN-γ) ELISpot was performed essentially as previously described (Draper et al., 2010), with the following modifications. Anti-human IFN-γ antibodies were used, as in previous *Aotus* ELISPOT experiments (Jordan-Villegas et al., 2011): mAb 1-D1-K for capture, and biotinylated mAb 7-B6-1 as secondary (both from Mabtech). Assays were performed from frozen PBMC with all samples tested at the same time. The final assay used 100,000 PBMC per well. PfRH5 peptides (20mers overlapping by 10 αα spanning the PfRH5 antigen sequence, *n*=50) were synthesised by Mimotopes Ltd, resuspended in DMSO to 100mg/mL, and pooled. PfAMA1 peptides (20mers overlapping by 10 αα spanning the bi-allelic PfAMA1 antigen sequence present in the ChAd63 vaccine) have previously been described (Sheehy et al., 2012a). Peptide pools for both PfRH5 and PfAMA1 were diluted in complete R10 medium (RPMI 1640, supplemented with 10% FCS, 4 mM L-glutamine, 100 U/mL

penicillin, 100 µg/mL streptomycin, 10 mM HEPES buffer, and 50 µg/mL gentamicin [all from Sigma]) and used at a final in-well concentration of 5µg/mL of each peptide with no more than 0.5% total DMSO. Wells containing 10µg/mL phytohaemagglutinin (PHA) and 20ng/mL staphylococcal enterotoxin B (SEB) (both from Sigma) were used as positive controls; negative control wells contained R10 with 0.5% DMSO only. Cells from each animal were tested in duplicate for PfRH5 and PfAMA1 peptide-containing wells and negative control wells, and in a single positive control well.

Intracellular Cytokine Staining

Antigen-specific IFN-γ production by T cells from frozen PBMC samples was assayed by an intracellular cytokine staining (ICS) assay based upon a previously published method (Draper et al., 2010). Briefly, PBMC were stimulated for 16 h in the presence of anti-human CD49d (BD Biosciences), brefeldin A (GolgiPlug, BD Biosciences), and monensin (Golgi Stop, BD Biosciences). The following stimulation conditions were included for each sample: i) positive control: 50 ng/mL of Phorbol 12-myristate 13-acetate (PMA) and 0.5 µg/mL of ionomycin (both from Sigma); ii) antigen-specific stimulation: either PfAMA1 or PfRH5 peptide pools, as described above for ELISpot; and iii) unstimulated control: 0.11% total DMSO in R10 media. Cells were stained the next day using Live/Dead Red stain (Invitrogen) and a cocktail of fluorochrome-conjugated monoclonal antibodies: phycoerythrin-Cy7- (PECy7) conjugated anti-human CD4 (clone SK3) and phycoerythrin- (PE) conjugated anti-human CD8α (clone RPA; both from BD Biosciences). After this surface staining, cells were fixed with Cytofix/Cytoperm (BD Biosciences) for 10 min at 4°C before intracellular staining in Permwash (BD Biosciences) with fluorescein isothiocyanate- (FITC) conjugated anti-human IFN-γ (clone B27, BD Biosciences). All antibody clones had previously been tested for specific recognition of their respective molecules in *Aotus* cells and titrated to identify optimal working concentrations. Stained cells were analyzed using a FACS Calibur instrument (BD Biosciences) and FlowJo v7.6 software (Tree Star Inc, USA). $IFN\gamma^+CD4^+$ and $IFN\gamma^+CD8^+$ cells were calculated as percentages of total live $CD4^+$ and total $CD8^+$ lymphocytes

respectively. Background responses in the unstimulated condition were subtracted from antigen-specific responses.

ELISA and CFCA

For PfAMA1 ELISAs, Nunc-Immuno Maxisorp plates were coated in PBS containing 2 μg/mL of the same recombinant PfAMA1 FVO protein used for immunization (Kennedy et al., 2002) and left over-night. For PfRH5 ELISAs, enzymatically mono-biotinylated PfRH5 protein was produced by transient transfection of suspension adapted and serum-free adapted HEK293E cells. The ELISA antigen encoded the version of the PfRH5 antigen expressed in the viral-vector vaccines (which lacks the CD4 d3+4 and His6 tags present in the protein vaccine). This sequence was amplified by PCR and ligated into a mammalian expression vector under the control of the human immediate-early CMV promoter (Sridhar et al., 2008) expressing the antigen fused to a C-terminal purification tag consisting of an AviTag biotin acceptor peptide followed by a StrepII tag, in tandem. The PfRH5 antigen cassette was preceded by an in-frame N-terminal human tissue plasminogen activator leader sequence (Draper et al., 2008) for the secretion of recombinant protein into the culture supernatant. The plasmid vector was amplified in DH5α *Escherichia coli* and purified using an EndoFree plasmid maxi kit (QIAGEN). Suspension cultures of HEK293E cells were transfected with DNA using the method of Durocher *et al.* (Durocher et al., 2002). Culture medium was Freestyle293 (Gibco) supplemented with 1% FBS. 84 h post-transfection, HEK293E cell culture supernatant was harvested, filtered through a 0.22μm polyethersulfone membrane (Millipore) and extensively buffer-exchanged with a 10kDa MWCO SnakeSkin dialysis membrane (Thermo) into PBS overnight at 4˚C. 100 μL protein supernatant was used to coat 96 well Nunc Immobilizer streptavidin-coated plates and left over-night.

The next day plates were washed 6x in PBS containing 0.05% Tween 20 (PBS/T) and blocked for 1h with Casein block solution (Pierce, UK). Plates were washed again, and then a standard plasma sample, test plasma, internal control and blank samples all diluted in Casein block solution were added to each

plate for 2 h according to published methodology (Miura et al., 2008; Sheehy et al., 2011). The standards for PfRH5 and PfAMA1 were prepared from a pool of Group B and E plasma (from the day 69 time-point) respectively. The pool was serially diluted on every plate to make a standard curve. Test plasma were diluted 1:300 and tested in duplicate wells. Plates were washed again, followed by addition for 1 h of alkaline phosphatase-conjugated rabbit anti-monkey IgG (whole molecule) (Sigma) diluted 1:5000 in Casein block solution. Plates were washed again and bound antibodies were detected by adding *p*-nitrophenylphosphate substrate (Sigma) diluted in diethanolamine buffer (Fisher Scientific, UK). Optical density was read at 405nm (OD_{405}) using an ELx800 microplate reader (BioTek, UK). The ELISA antibody unit (AU) value of the standard was assigned as the reciprocal of the dilution giving an OD_{405} of 1.0 in the standardized assay. The OD_{405} of individual test samples was converted into AU by using the standard curve and Gen5 ELISA software v1.10 (BioTek, UK). If the OD_{405} of test plasma was too high to read off the linear part of the curve, the assays were repeated, testing plasma at a higher dilution (typically 1:1000 – 1:10000). The OD-based ELISA AU results were converted to µg/mL using the results of calibration-free concentration analyses (CFCA), as described below.

For $PfMSP1_{19}$ ELISAs, the production of recombinant glutathione *S*-transferase (GST) protein and $PfMSP1_{19}$-GST fusion protein (QKNG allele) has been previously described (Goodman et al., 2010). Plates were coated with 2µg/mL protein in PBS and left over-night. Plasma were diluted 1:300, added in duplicate and the ELISA method otherwise followed as above. Plates were developed, and the average OD_{405} reading is reported for each sample. All plasma tested against the GST control protein showed no detectable response above background levels (data not shown).

CFCA analyses were performed with a method similar to that previously described (Williams et al., 2012), using a Biacore T200 machine, a Biotin CAP chip, and T200 control and evaluation software (all from GE Lifesciences, Amersham, UK). The PfRH5 antigen used for CFCA had the same sequence as

the protein vaccine and was produced by transient transfection of HEK293E cells, as previously described (Crosnier et al., 2011). The PfAMA1 antigen used for CFCA was produced as described above, by generating a plasmid encoding the FVO PfAMA1 sequence (identical to both the protein and ChAd63 vaccines), codon-optimized for mammalian expression (Draper et al., 2010), and with C-terminal Avitag and StrepII tags. Both antigens were enzymatically mono-biotinylated by co-transfection of the cultures with a plasmid encoding BirA (Bushell et al., 2008), then dialysed extensively against PBS prior to CFCA. Plasma pools were prepared from Group A (control), Group D (PfRH5-immunized) and Group E (PfAMA1-immunized) samples obtained on day 69 (DoC). Three replicate dilutions of each (1:1250 in running buffer) were prepared. Mass-transport limited binding conditions were obtained by capturing a minimum of 800 response units (RU) of antigen on flow cell 2. The chip was regenerated with the manufacturer's supplied regeneration and CAP reagents and fresh antigen prior to each application of antibody; variation in the level of antigen capture between cycles was typically <2%.

As shown in Figure S3A-C, antigen-specific antibody binding was measured by double reference subtraction, firstly of binding to a flow cell coated only with the biotin capture reagent, and secondly of the binding of the control (Group A) samples from the immune (Group D or E) sample. Initial rates of antigen-specific binding at 5 µL/min and 100 µL/min were measured and compared to permit measurement of concentration and the level of mass-transport limitation. The binding model used a molecular weight of 150 kDa for IgG and a diffusion coefficient of IgG under the test conditions (37°C, running buffer) of 5.5×10^{-11} m^2/s[25].

All results reported were within the instrument manufacturer's recommended quality control parameters, namely initial binding rates in the range 0.3RU to 15 RU/s at 5 µL/min flow, and QC ratio >0.13 (reflecting adequate mass transport limitation for concentration estimation).

The CFCA-measured antigen-specific antibody concentrations in the Group D and Group E pools were combined with the known ELISA AU measurements for the same samples to derive an AU-to-µg/mL conversion factor which was applied to express other ELISA results in terms of µg/mL units.

Assays of Growth Inhibitory Activity (GIA)

Assays of GIA were performed at the PATH-MVI GIA reference laboratory, NIAID, NIH, using a previously published method (Miura et al., 2009). Total IgG was purified using protein G (Pierce). Human O+ RBC and FVO strain *P. falciparum* parasites were used. A single-lifecycle assay was performed, followed by growth quantification by colorimetric detection of parasite lactate dehydrogenase. All plates included controls as follows: no-parasite (zero growth); no-antibody (maximum growth); rabbit-anti PfAMA1 IgG (positive control standard at two concentrations, which performed comparably to previous assays with this parasite line). All samples were tested in triplicate wells. Duplicate experiments were performed with total IgG at a final in-well concentration of 2.5mg/mL; in the second experiment, GIA was further measured with 3-fold serial dilutions of total IgG concentration down to a minimum of 0.03mg/mL.

For each sample achieving >50% GIA at 2.5mg/mL, total IgG GIA EC_{50} was calculated in terms of total IgG concentration in the well by linear interpolation (identification of the value on a $\log_{10}$-transformed x-axis at the intercept of a straight line between the IgG concentrations achieving immediately in excess of and below 50% GIA).

The total IgG concentration in each plasma sample was measured using Protein A biosensors on a Fortebio Blitz instrument (ForteBio, Menlo Park, USA). A standard curve was generated using Protein G purified *Aotus* IgG and the Create Standard Curve module in the Blitz Pro Data Analysis software. Sample IgG concentration was then quantified relative to this standard curve using the manufacturer's recommended protocol for the Quantitate Sample module. For each animal

achieving >50% GIA at 2.5mg/mL, the "GIA_{50} titer" was then calculated by dividing the plasma total IgG concentration by the total IgG GIA EC_{50}.

Analyses and Statistics

Throughout, all reported *P* values are for two-tailed tests.

Efficacy analyses

The following vaccine efficacy endpoints were recorded, as used in a previous *Aotus* – *P. falciparum* challenge study (Lyon et al., 2008) and a recent study of *P. knowlesi* infection of rhesus macaques (Mahdi Abdel Hamid et al., 2011).

1. Ordinally-ranked treatment status (treatment for parasitemia [TxP]; treatment for anaemia [TxA]; no treatment).
2. As a continuous variable, $\log_{10}$(cumulative parasitemia) (LCP) up to the day on which the first animal in the study required treatment (in this case, day 10).

The Group A sham vaccinated animals served as the protocol pre-specified infectivity controls, in order to confirm consistent infection by the FVO parasite inoculum and its appropriate adaptation to growth in *Aotus*. For Group B (Freund's adjuvant), no adjuvant-matched control group was thus included in the study, but historical control data were available from 15 previously-published studies in which *Aotus nancymaae* were subjected to FVO-strain challenge after receiving Freund's adjuvant without a blood-stage antigen (Table S1). Of a total of 55 such animals, 48 required treatment for uncontrolled parasitemia, while 7 required treatment for anaemia. Kendall's tau-b was used to test a null hypothesis of equivalent outcome between Group B and historical Freund's control animals using the ordinally ranked outcome data. As a secondary efficacy outcome measure for this group (using non-adjuvant-

matched control data from the current study), LCP was compared between Groups B and A by Mann-Whitney test.

For Groups C and E, Group A was an adjuvant-matched control group. No ChAd63-MVA control group was included for Group D, and there is no direct historical control available for these animals. Extensive experience from rodent malaria studies (using three *Plasmodium* spp.) and Phase IIa *P. falciparum* controlled human malaria infection (CHMI) trials suggests that MVA immunization two weeks prior to infection does not confer any non-specific inhibitory effect upon blood-stage malaria parasite growth (Biswas et al., 2012; Draper et al., 2009; Goodman et al., 2013; Sheehy et al., 2012b). There was also no non-specific effect of vaccination with COPAK (a poxvirus similar to the MVA) upon the course of *P. knowlesi* parasitemia in rhesus macaques (Weiss et al., 2007). Group A was thus the most relevant available comparator for Group D. The protocol-specified primary analysis of efficacy in Groups C, D and E was therefore comparison of LCP in each Group to Group A by Mann-Whitney test with Bonferroni correction for multiple comparison.

A post-hoc secondary analysis of efficacy in terms of effect upon time to treatment was performed using a Mann-Whitney test with Bonferroni correction for multiple comparison, comparing each of Groups B, C, D and E to Group A (a log-rank survival analysis was felt inappropriate in view of doubt regarding the validity of its proportional hazards assumption).

Analyses of association between immunological parameters and outcome

The majority of immunological parameters were non-normally distributed and thus, unless further detailed below, analyses of association between immunological parameters and continuous outcome variables were performed by Spearman's rank correlation.

The protocol-specified primary analysis for a correlate of protection, in the event that GIA EC_{50} data could not be estimated for every animal (as was the case here for a number of the animals in Groups C and E), was examination of the correlation between GIA at a fixed total IgG concentration and *in vivo* growth inhibition (IVGI; the percentage reduction in the parasite multiplication rate (PMR) in each animal relative to the mean in the control group, as has been previously described (Mahdi Abdel Hamid et al., 2011)).

To calculate IVGI, initial *in vivo* PMR was estimated for all animals. We have recently reported that a simple linear model performs comparably to more complex parasite growth models in estimating PMR in CHMI trials (Douglas et al., 2013), and used a similar approach here. We sought to achieve accurate estimation of the initial PMR by using all microscopically-quantified parasitemias up to day 10 post-challenge, or the 4th day of microscopic patency (whichever was later). We felt this approach offered an appropriate balance between excessive reliance on very early and inaccurately quantified low-level parasitemia measurements and the likelihood of progressively altering PMR later in infection due to the development of secondary infection-induced (as opposed to vaccine-induced) immune responses. The initial parasitemia in p/µL was calculated by dividing the inoculum (10,000) by each animal's blood volume, corrected for weight (estimated at 70mL/kg). For each animal, the initial parasitemia and this subset of the observed parasitemia data were $\log_{10}$ transformed and the slope, m, of a linear regression line through these points was calculated. PMR (fold per 48 hours) was then calculated as 10^{2m}. Finally, i*n vivo* growth inhibition (IVGI) was estimated as:

$$100 * \left(1 - \left[\frac{PMR_{individual}}{PMR_{Group\ A\ mean}}\right]\right)$$

For animals in Group B that did not become microscopically patent at any point (and the one animal in Group B in which a single parasite was seen on day 3 only), PMRs were estimated by arbitrarily

assigning parasitemias of 400p/µL, around the threshold of detection, on day C+28 of the study (the day on which they were treated).

In view of the fact that group allocation was clearly associated with challenge outcome, a further analysis was undertaken to establish that GIA was independently predictive of challenge outcome. Linear regression models of the relationship of IVGI with group only (bivariate model) and of IVGI with group plus GIA at 2.5mg/mL (multivariate model) were compared by likelihood-ratio test, using Stata 12.0 (StataCorp, USA).

The relationship between antigen-specific antibody concentration and IVGI (Figure 3D) was assessed by fitting a variable slope dose-response curve using the equation:

$$\% \text{ IVGI} = 100 / (1+10\hat{}((\log10EC_{50} - \log10[IgG]) * \text{Hill Slope}))$$

The curve was constrained to a maximal level of IVGI of 100% and was fitted using Prism v5.03 software (GraphPad Software). Residuals were not significantly non-normal (Shapiro-Wilk, *P*=0.10), nor was a systematic pattern evident upon visual examination of a residual plot.

SUPPLEMENTAL REFERENCES

Baum, J., Chen, L., Healer, J., Lopaticki, S., Boyle, M., Triglia, T., Ehlgen, F., Ralph, S.A., Beeson, J.G., and Cowman, A.F. (2009). Reticulocyte-binding protein homologue 5 - an essential adhesin involved in invasion of human erythrocytes by Plasmodium falciparum. Int J Parasitol *39*, 371-380.

Biswas, S., Dicks, M.D., Long, C.A., Remarque, E.J., Siani, L., Colloca, S., Cottingham, M.G., Holder, A.A., Gilbert, S.C., Hill, A.V.*, et al.* (2011). Transgene Optimization, Immunogenicity and In Vitro Efficacy of Viral Vectored Vaccines Expressing Two Alleles of Plasmodium falciparum AMA1. PLoS One *6*, e20977.

Biswas, S., Spencer, A.J., Forbes, E.K., Gilbert, S.C., Holder, A.A., Hill, A.V., and Draper, S.J. (2012). Recombinant Viral-Vectored Vaccines Expressing Plasmodium chabaudi AS Apical Membrane Antigen 1: Mechanisms of Vaccine-Induced Blood-Stage Protection. J Immunol *188*, 5041-5053.

Bushell, K.M., Sollner, C., Schuster-Boeckler, B., Bateman, A., and Wright, G.J. (2008). Large-scale screening for novel low-affinity extracellular protein interactions. Genome Res *18*, 622-630.

Bustamante, L.Y., Bartholdson, S.J., Crosnier, C., Campos, M.G., Wanaguru, M., Nguon, C., Kwiatkowski, D.P., Wright, G.J., and Rayner, J.C. (2013). A full-length recombinant Plasmodium falciparum PfRH5 protein induces inhibitory antibodies that are effective across common PfRH5 genetic variants. Vaccine *31*, 373-379.

Crosnier, C., Bustamante, L.Y., Bartholdson, S.J., Bei, A.K., Theron, M., Uchikawa, M., Mboup, S., Ndir, O., Kwiatkowski, D.P., Duraisingh, M.T.*, et al.* (2011). Basigin is a receptor essential for erythrocyte invasion by Plasmodium falciparum. Nature *480*, 534-537.

Crosnier, C., Staudt, N., and Wright, G.J. (2010). A rapid and scalable method for selecting recombinant mouse monoclonal antibodies. BMC biology *8*, 76.

Deans, J.A., Alderson, T., Thomas, A.W., Mitchell, G.H., Lennox, E.S., and Cohen, S. (1982). Rat monoclonal antibodies which inhibit the in vitro multiplication of Plasmodium knowlesi. Clin Exp Immunol *49*, 297-309.

Douglas, A.D., Edwards, N.J., Duncan, C.J., Thompson, F.M., Sheehy, S.H., O'Hara, G.A., Anagnostou, N., Walther, M., Webster, D.P., Dunachie, S.J.*, et al.* (2013). Comparison of Modeling Methods to Determine Liver-to-blood Inocula and Parasite Multiplication Rates During Controlled Human Malaria Infection. J Infect Dis *208*, 340-345.

Douglas, A.D., Williams, A.R., Illingworth, J.J., Kamuyu, G., Biswas, S., Goodman, A.L., Wyllie, D.H., Crosnier, C., Miura, K., Wright, G.J.*, et al.* (2011). The blood-stage malaria antigen PfRH5 is susceptible to vaccine-inducible cross-strain neutralizing antibody. Nat Commun *2*, 601.

Draper, S.J., Biswas, S., Spencer, A.J., Remarque, E.J., Capone, S., Naddeo, M., Dicks, M.D.J., Faber, B.W., de Cassan, S.C., Folgori, A.*, et al.* (2010). Enhancing blood-stage malaria subunit vaccine immunogenicity in rhesus macaques by combining adenovirus, poxvirus, and protein-in-adjuvant vaccines. J Immunol *185*, 7583-7595.

Draper, S.J., Goodman, A.L., Biswas, S., Forbes, E.K., Moore, A.C., Gilbert, S.C., and Hill, A.V. (2009). Recombinant viral vaccines expressing merozoite surface protein-1 induce antibody- and T cell-mediated multistage protection against malaria. Cell Host Microbe *5*, 95-105.

Draper, S.J., Moore, A.C., Goodman, A.L., Long, C.A., Holder, A.A., Gilbert, S.C., Hill, F., and Hill, A.V. (2008). Effective induction of high-titer antibodies by viral vector vaccines. Nat Med *14*, 819-821.

Durocher, Y., Perret, S., and Kamen, A. (2002). High-level and high-throughput recombinant protein production by transient transfection of suspension-growing human 293-EBNA1 cells. Nucleic Acids Res *30*, E9.

Dutta, S., Sullivan, J.S., Grady, K.K., Haynes, J.D., Komisar, J., Batchelor, A.H., Soisson, L., Diggs, C.L., Heppner, D.G., Lanar, D.E.*, et al.* (2009). High antibody titer against apical membrane antigen-1 is required to protect against malaria in the Aotus model. PLoS One *4*, e8138.

Freeman, R.R., Trejdosiewicz, A.J., and Cross, G.A. (1980). Protective monoclonal antibodies recognising stage-specific merozoite antigens of a rodent malaria parasite. Nature *284*, 366-368.

Goodman, A.L., Epp, C., Moss, D., Holder, A.A., Wilson, J.M., Gao, G.P., Long, C.A., Remarque, E.J., Thomas, A.W., Ammendola, V.*, et al.* (2010). New candidate vaccines against blood-stage Plasmodium falciparum malaria: prime-boost immunization regimens incorporating human and simian adenoviral vectors and poxviral vectors expressing an optimized antigen based on merozoite surface protein 1. Infect Immun *78*, 4601-4612.

Goodman, A.L., Forbes, E.K., Williams, A.R., Douglas, A.D., de Cassan, S.C., Bauza, K., Biswas, S., Dicks, M.D., Llewellyn, D., Moore, A.C.*, et al.* (2013). The utility of Plasmodium berghei as a rodent model for anti-merozoite malaria vaccine assessment. Sci Rep *3*, 1706.

Jordan-Villegas, A., Perdomo, A.B., Epstein, J.E., Lopez, J., Castellanos, A., Manzano, M.R., Hernandez, M.A., Soto, L., Mendez, F., Richie, T.L.*, et al.* (2011). Immune responses and protection of Aotus monkeys immunized with irradiated Plasmodium vivax sporozoites. Am J Trop Med Hyg *84*, 43-50.

Kennedy, M.C., Wang, J., Zhang, Y., Miles, A.P., Chitsaz, F., Saul, A., Long, C.A., Miller, L.H., and Stowers, A.W. (2002). In vitro studies with recombinant Plasmodium falciparum apical membrane antigen 1 (AMA1): production and activity of an AMA1 vaccine and generation of a multiallelic response. Infect Immun *70*, 6948-6960.

Lyon, J.A., Angov, E., Fay, M.P., Sullivan, J.S., Girourd, A.S., Robinson, S.J., Bergmann-Leitner, E.S., Duncan, E.H., Darko, C.A., Collins, W.E.*, et al.* (2008). Protection induced by Plasmodium falciparum MSP1(42) is strain-specific, antigen and adjuvant dependent, and correlates with antibody responses. PLoS ONE *3*, e2830.

Mahdi Abdel Hamid, M., Remarque, E.J., van Duivenvoorde, L.M., van der Werff, N., Walraven, V., Faber, B.W., Kocken, C.H., and Thomas, A.W. (2011). Vaccination with Plasmodium knowlesi AMA1 formulated in the novel adjuvant co-vaccine HT protects against blood-stage challenge in rhesus macaques. PLoS One *6*, e20547.

Miura, K., Orcutt, A.C., Muratova, O.V., Miller, L.H., Saul, A., and Long, C.A. (2008). Development and characterization of a standardized ELISA including a reference serum on each plate to detect antibodies induced by experimental malaria vaccines. Vaccine *26*, 193-200.

Miura, K., Zhou, H., Diouf, A., Moretz, S.E., Fay, M.P., Miller, L.H., Martin, L.B., Pierce, M.A., Ellis, R.D., Mullen, G.E.*, et al.* (2009). Anti-apical-membrane-antigen-1 antibody is more effective than anti-42-kilodalton-merozoite-surface-protein-1 antibody in inhibiting plasmodium falciparum growth, as determined by the in vitro growth inhibition assay. Clin Vaccine Immunol *16*, 963-968.

Orubu, T., Alharbi, N.K., Lambe, T., Gilbert, S.C., and Cottingham, M.G. (2012). Expression and cellular immunogenicity of a transgenic antigen driven by endogenous poxviral early promoters at their authentic loci in MVA. PLoS One *7*, e40167.

Sanderson, F., Andrews, L., Douglas, A.D., Hunt-Cooke, A., Bejon, P., and Hill, A.V. (2008). Blood-stage challenge for malaria vaccine efficacy trials: a pilot study with discussion of safety and potential value. Am J Trop Med Hyg *78*, 878-883.

Sauerwein, R.W., Roestenberg, M., and Moorthy, V.S. (2011). Experimental human challenge infections can accelerate clinical malaria vaccine development. Nat Rev Immunol *11*, 57-64.

Sheehy, S.H., Douglas, A.D., and Draper, S.J. (2013). Challenges of assessing the clinical efficacy of asexual blood-stage Plasmodium falciparum malaria vaccines. Hum Vaccin Immunother *9*, 1831-1840.

Sheehy, S.H., Duncan, C.J., Elias, S.C., Biswas, S., Collins, K.A., O'Hara, G.A., Halstead, F.D., Ewer, K.J., Mahungu, T., Spencer, A.J.*, et al.* (2012a). Phase Ia Clinical Evaluation of the Safety and Immunogenicity of the Plasmodium falciparum Blood-Stage Antigen AMA1 in ChAd63 and MVA Vaccine Vectors. PLoS One *7*, e31208.

Sheehy, S.H., Duncan, C.J., Elias, S.C., Choudhary, P., Biswas, S., Halstead, F.D., Collins, K.A., Edwards, N.J., Douglas, A.D., Anagnostou, N.A.*, et al.* (2012b). ChAd63-MVA-vectored Blood-stage Malaria Vaccines Targeting MSP1 and AMA1: Assessment of Efficacy Against Mosquito Bite Challenge in Humans. Mol Ther *20*, 2355-2368.

Sheehy, S.H., Duncan, C.J., Elias, S.C., Collins, K.A., Ewer, K.J., Spencer, A.J., Williams, A.R., Halstead, F.D., Moretz, S.E., Miura, K.*, et al.* (2011). Phase Ia Clinical Evaluation of the Plasmodium falciparum Blood-stage Antigen MSP1 in ChAd63 and MVA Vaccine Vectors. Mol Ther *19*, 2269-2276.

Sridhar, S., Reyes-Sandoval, A., Draper, S.J., Moore, A.C., Gilbert, S.C., Gao, G.P., Wilson, J.M., and Hill, A.V. (2008). Single-dose protection against Plasmodium berghei by a simian adenovirus vector using a human cytomegalovirus promoter containing intron A. J Virol *82*, 3822-3833.

Stowers, A.W., Cioce, V., Shimp, R.L., Lawson, M., Hui, G., Muratova, O., Kaslow, D.C., Robinson, R., Long, C.A., and Miller, L.H. (2001). Efficacy of two alternate vaccines based on Plasmodium falciparum merozoite surface protein 1 in an Aotus challenge trial. Infect Immun *69*, 1536-1546.

Stowers, A.W., Kennedy, M.C., Keegan, B.P., Saul, A., Long, C.A., and Miller, L.H. (2002). Vaccination of monkeys with recombinant Plasmodium falciparum apical membrane antigen 1 confers protection against blood-stage malaria. Infect Immun *70*, 6961-6967.

Thera, M.A., Doumbo, O.K., Coulibaly, D., Laurens, M.B., Ouattara, A., Kone, A.K., Guindo, A.B., Traore, K., Traore, I., Kouriba, B.*, et al.* (2011). A field trial to assess a blood-stage malaria vaccine. N Engl J Med *365*, 1004-1013.

Weiss, W.R., Kumar, A., Jiang, G., Williams, J., Bostick, A., Conteh, S., Fryauff, D., Aguiar, J., Singh, M., O'Hagan, D.T.*, et al.* (2007). Protection of rhesus monkeys by a DNA prime/poxvirus boost malaria vaccine depends on optimal DNA priming and inclusion of blood stage antigens. PLoS ONE *2*, e1063.

Williams, A.R., Douglas, A.D., Miura, K., Illingworth, J.J., Choudhary, P., Murungi, L.M., Furze, J.M., Diouf, A., Miotto, O., Crosnier, C.*, et al.* (2012). Enhancing Blockade of Plasmodium falciparum Erythrocyte Invasion: Assessing Combinations of Antibodies against PfRH5 and Other Merozoite Antigens. PLoS Pathog *8*, e1002991.
